# PorV is an Outer Membrane Shuttle Protein for the Type IX Secretion System

**DOI:** 10.1038/s41598-017-09412-w

**Published:** 2017-08-18

**Authors:** Michelle D. Glew, Paul D. Veith, Dina Chen, Dhana G. Gorasia, Ben Peng, Eric C. Reynolds

**Affiliations:** Oral Health Cooperative Research Centre, Melbourne Dental School, Bio21 Institute, The University of Melbourne, Melbourne, Australia

## Abstract

*Porphyromonas gingivalis* is a keystone pathogen associated with chronic periodontitis. Major virulence factors named gingipains (cysteine proteinases, RgpA, RgpB and Kgp) are secreted via the Type IX Secretion System (T9SS). These, together with approximately 30 other proteins, are secreted to the cell surface and anchored to the outer membrane by covalent modification to anionic lipopolysaccharide (A-LPS) via the novel Gram negative sortase, PorU. PorU is localised on the cell surface and cleaves the C-terminal domain signal (CTD) of T9SS substrates and conjugates their new C-termini to A-LPS. A 440 kDa-attachment complex was identified in the wild-type (WT) comprising of PorU:PorV:PorQ:PorZ. In mutant strains, sub-complexes comprising PorU:PorV or PorQ:PorZ were also identified at smaller native sizes suggesting that PorU and PorZ are anchored to the cell surface via interaction with the PorV and PorQ outer membrane proteins, respectively. Analysis of *porU* mutants and a CTD cleavage mutant revealed accumulation of immature T9SS substrates in a PorV-bound form. Quantitative label-free proteomics of WT whole cell lysates estimated that the proportion of secretion channels:attachment complexes:free PorV:T9SS substrates was 1:6:110:2000 supporting a role for PorV as a shuttle protein delivering secreted proteins to the attachment complex for CTD signal cleavage and A-LPS modification.

## Introduction

*Porphyromonas gingivalis* is considered to be a keystone pathogen associated with chronic periodontitis^1^. This is a progressive inflammatory disease of the tooth’s supporting tissues which results in destruction of those tissues and ultimate tooth loss. The major virulence factors of *P. gingivalis* include the cysteine proteinases known as gingipains^2–5^. The gingipains comprise two Arg-specific proteinases, RgpA and RgpB and one lysine-specific, Kgp. RgpA and Kgp are synthesised as polyprotein precursors which are proteolytically processed on the cell surface to yield their respective catalytic (cat) domains and several adhesin domains numbered A1 to A5^2, 6^. Each gingipain has a leader sequence followed by an N-terminal pro-domain, which is cleaved to activate the cat domain, and a conserved C-terminal domain (CTD). RgpB does not have adhesin domains and during maturation, the pro domain of pro-RgpB (RgpB [II]) is cleaved in two steps producing RgpB [III] and RgpB [IV] respectively, and finally the CTD is also cleaved^7–9^.

Gingipains, together with approximately 30 other proteins, are secreted to the cell surface and anchored to the outer membrane (OM) by covalent attachment to an anionic lipopolysaccharide (A-LPS)^6, 10^. This secretion and attachment is performed by the Type IX Secretion System (T9SS) that has been found only in members of the *Bacteroidetes/Chlorobi* phyla^11–14^. T9SS substrates require an N-terminal leader sequence for transport across the inner membrane (IM) by the sec translocon and a conserved T9SS-specific CTD required for secretion across the OM and covalent linkage to A-LPS^10, 15, 16^. Many proteins involved in the T9SS have been identified and include proteins required for A-LPS biosynthesis and transport^17^, 14 components required for protein secretion and attachment to A-LPS (PorK, PorL, PorM, PorN, PorP, PorQ, PorT, PorU, PorV, PorW, PorZ, Sov, PG0534 and PG1058)^11, 18–23^, and regulatory proteins, (PorY, PorX, GppX and SigP)^11, 24–26^.

The structural and functional characterisation of the T9SS is still in its infancy. PorK and PorN form very large ring-shaped complexes of 50 nm diameter that are associated with the OM^27^ and may comprise part of a trans-envelope secretion apparatus due to interactions with inner membrane components PorL and PorM^28^. PG0026 (PorU) which contains a gingipain-like protease domain is responsible for cleaving the CTD^7^ and linking the new C-terminus to A-LPS via a sortase-like transpeptidase mechanism^10^. In *P. gingivalis*, the CTD cleavage site has been identified for many CTD proteins^6, 7^. The cleavage site occurs in a disordered linker region between the Ig-like and CTD domains consistent with it being flexible and exposed for PorU cleavage^29^. Disruption of this cleavage site by deletion of two or six residues causes accumulation of immature RgpB in both the membrane and soluble fractions indicating that CTD cleavage and A-LPS modification were impaired^30^.

PorU has an uncleavable CTD^7^ and locates to the surface of the OM by non-covalent interaction with PorV, an outer membrane β-barrel protein^8, 31^. We have previously found the PorU sortase to be present with PorV in two sized complexes^8^. The larger native 480 kDa complex occurred in the WT but the lower sized 330 kDa complex, comprising only PorU and PorV, was found in a strain that did not synthesise A-LPS. We proposed therefore that other proteins may be present in the WT PorU sortase complex^8^. Like PorU, PG1604 retains its CTD, is not A-LPS modified and is predicted to be a secreted component of the T9SS^6, 22^. Very recently PG1604, named PorZ, was experimentally demonstrated to be a T9SS component and its crystal structure elucidated^23^.

CTD proteins in *porU* and *porV* mutants have uncleaved CTD and are exposed on the surface only in *porU* ^7^. It was concluded that while secretion is completely blocked in the absence of PorV, some secretion occurs in *porU*, suggesting that the secreted proteins may be temporarily anchored to the OM via interaction with an outer membrane protein^7^. In this study, we have characterised the attachment complex to include two new members, PorZ and PorQ. Additionally, we demonstrate that PorV is the component responsible for temporarily anchoring the T9SS substrates to the outer membrane prior to their conjugation to A-LPS. We therefore propose that PorV functions as a novel outer membrane shuttle protein by collecting newly secreted T9SS substrates and delivering them to the attachment complex.

## Results

### The attachment complex comprises PorU, PorV, PorZ and PorQ

**Figure 1:**
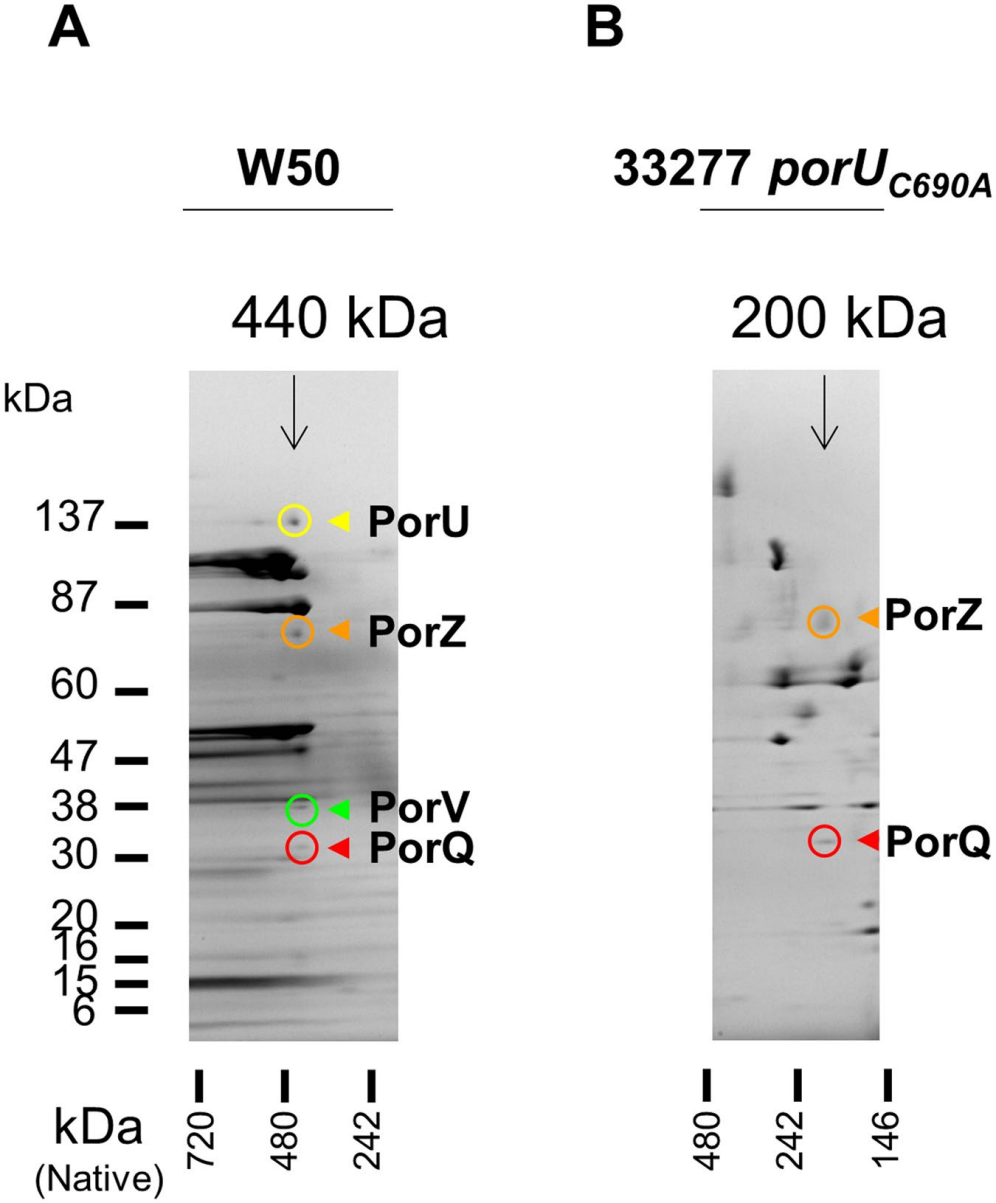
2D BN-PAGE analysis of OMV lysates reveals components of the sortase (PorU) attachment complex. Coomassie stained 2D BN-PAGE analyses for (**A**) large scale glycerol gradient fraction F17 of crude W50 OMV lysate, (**B**) glycerol gradient fraction F6 of W50 *porU*_*C690A*_ OMV lysate. Positions of PorU, PorZ, PorV and PorQ identified by MS are indicated by coloured circles and arrowheads. Native size of complexes are shown above. See Supplementary Fig. S1 and Supplementary Table S1 for spot numbers and MS data.

To address the possibility that other proteins were present in the PorV:PorU complex, WT OMVs were fractionated by a scaled-up glycerol gradient centrifugation protocol. The fractions containing the highest amount of PorU (F15–F17) were further analysed by 2D BN-PAGE (Fig. 1A and Supplementary Fig. S1). Four spots were found to co-align at a native size of 440 kDa and mass spectrometry (MS) analysis identified them as PorU, PorZ, PorV and PorQ, suggesting all four proteins constitute a complex (Supplementary Fig. S1 and Supplementary Table S1). The molar ratios of these components was approximately 1:1:3:1, respectively, suggesting that PorV was in excess (Supplementary Table S2). OMVs from the *porU* catalytic mutant, 33277 *porU*_*C690A*_, were also analyzed by 2D BN-PAGE and MS (Fig. 1B, Supplementary Fig. S1 and Supplementary Table S1). PorZ and PorQ were approximately equimolar (Supplementary Table S2) and aligned at a much smaller native size of 200 kDa suggesting that these two proteins were interacting. Previously, a complex comprising only PorU and PorV was identified in an A-LPS^−^ mutant^8^ and densitometry analysis estimated a molar ratio of 1:1.5 (Supplementary Table S2). The absolute stoichiometry for the subcomplexes appear to be approximately 2:2, however, the WT complex mass equates to a stoichiometry between [1:1:3:1] and [2:2:6:2] for PorU:PorZ:PorV:PorQ. The absolute stoichiometry of these various complexes are difficult to estimate since the migration of membrane proteins in BN-PAGE can differ from soluble standards^32^.

### PorZ and PorQ are required for CTD cleavage and attachment to A-LPS

**Figure 2:**
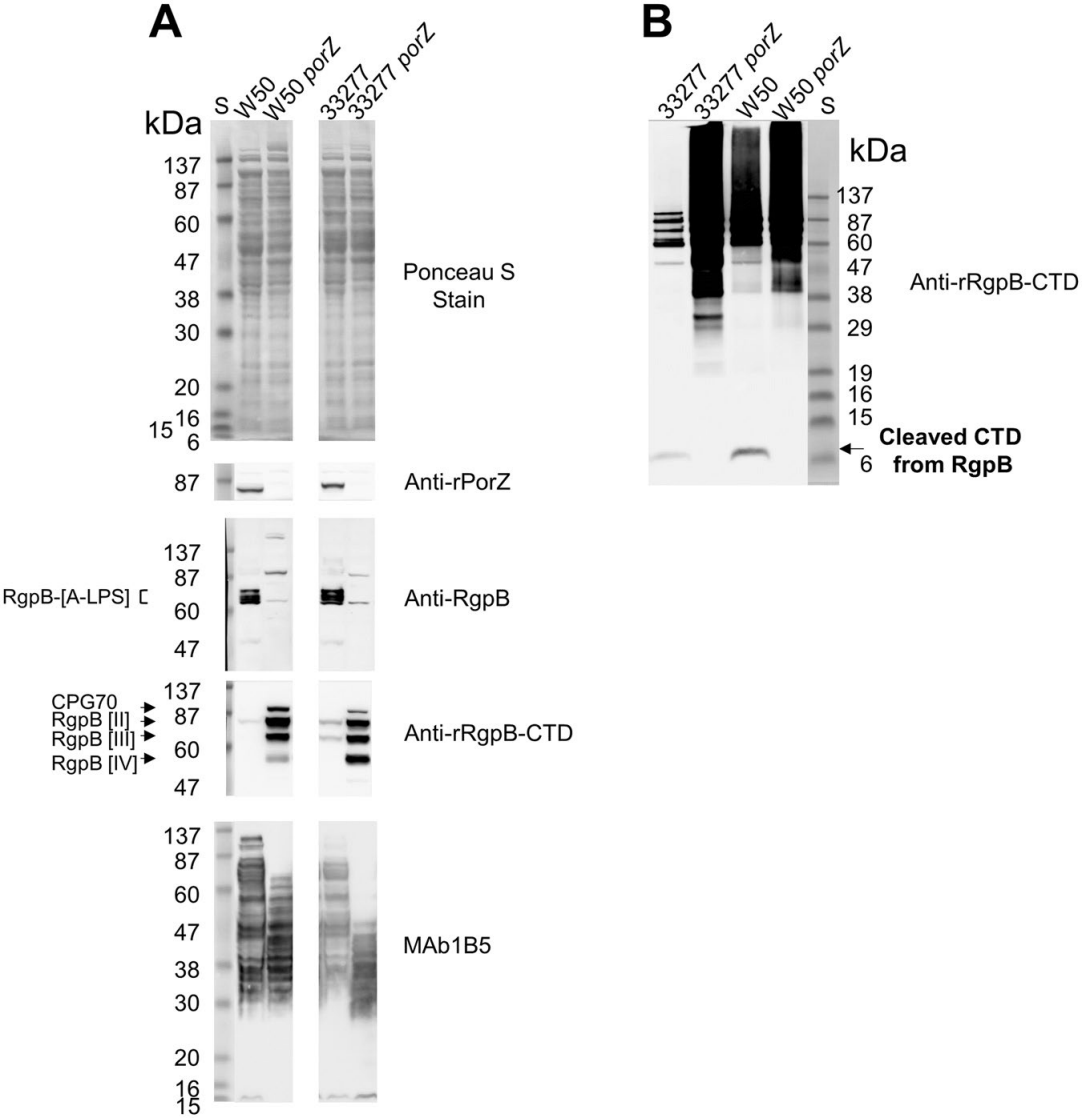
Characterisation of *porZ* mutants by Western blot. (**A**) Ponceau S stained SDS PAGE and Western blots of whole cell lysates from *porZ* mutants and parental WT strains. Equivalent amounts of log-phase cultures were compared. Mouse antisera or monoclonal antibody used are shown on right hand side of respective Western blot. (**B**) Anti-rRgpB-CTD Western blot of TCA-precipitated filtered and clarified culture fluid (CCFF, 1 ml) from indicated strains subjected to reducing SDS-PAGE using MES as running buffer. All samples were from cells grown to an OD_650nm_ of approximately 0.8. S, protein standard Prestained Benchmark Protein Ladder.

**Figure 3:**
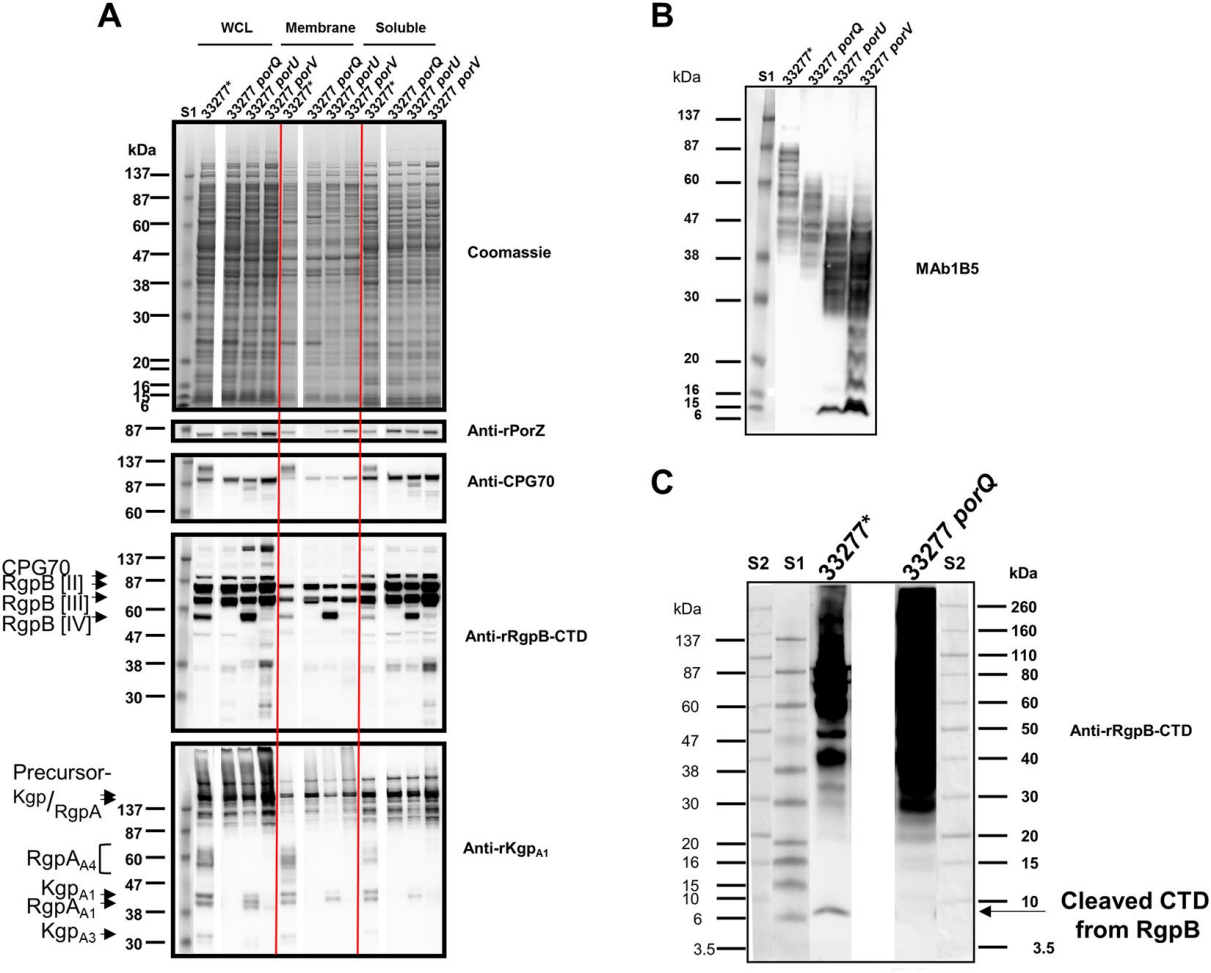
Characterisation of *porQ* mutant by Western blot. (**A**) Coomassie stained SDS PAGE and Western blots of *P. gingivalis* strain culture fractions. Equivalent amounts of log-phase culture fractions were compared. Mouse antisera used are shown on right hand side of respective Western blot. (**B**) Equivalent amounts of log-phase whole cell lysates subjected to MAb1B5 Western blot. (**C**) Anti-rRgpB-CTD Western blot of TCA-precipitated filtered and clarified culture fluid (CCFF, 1 ml) from indicated strains subjected to reducing SDS-PAGE using MES as running buffer. All samples were from cells grown to an OD_650nm_ of approximately 0.8. S1, protein standard Prestained Benchmark Protein Ladder. S2, Ponceau S stained protein standard Novex Sharp. White spaces between strain 33277* and mutant samples represents removal of irrelevant data.

The *porZ* gene was deleted in both W50 and ATCC 33277 backgrounds and found not to produce pigmentation (Supplementary Fig. S2). Western blot confirmed the absence of PorZ in both *porZ* mutants (Fig. 2A, anti-rPorZ). Both *porZ* mutants produced only immature forms of RgpB that were not covalently bound to A-LPS, similar to previous findings^23^ (Fig. 2A, anti-RgpB). There was also an accumulation of the 56 kDa pro-less form of RgpB [IV] (Fig. 2A, anti-rRgpB-CTD) similar to the *porU* mutant (Fig. 3A). Additionally, lower molecular weight (MW) forms of A-LPS were found in both *porZ* mutants indicative of unconjugated A-LPS (Fig. 2A, MAb1B5).

Western blot analysis indicated that the T9SS substrate CPG70 was present in both the ~90 kDa pro-form and the higher MW A-LPS modified form in WT, but only the latter preferentially partitioned to the membrane fraction, while *porQ* and the other T9SS mutants, *porU* and *porV*, only produced pro-form (Fig. 3A, anti-CPG70). Similarly, in WT, the gingipains Kgp and RgpA were present as both precursor forms (>150 kDa) and fully processed forms including A1 adhesins (39–44 kDa) and A-LPS modified RgpA_A4_ (~55–65 kDa) (Fig. 3A, anti-rKgp_A1_). In contrast, the T9SS mutants, *porQ*, *porU* and *porV*, possessed predominantly precursor forms of Kgp and RgpA, RgpA_A4_ was not modified with A-LPS and only *porU* exhibited proteolytic processing (Fig. 3A, anti-rKgp_A1_). Interestingly, the processing of RgpB in *porQ* was similar to *porV* with the production of RgpB pro-forms [II] and [III] but no pro-less [IV] (Fig. 3A, anti-rRgpB-CTD). Like *porU* and *porV*, *porQ* exhibited a lower MW profile of A-LPS positive bands suggesting unconjugated A-LPS (Fig. 3B).

Because covalent linkage of A-LPS was absent in both the *porZ* and *porQ* mutants, CTD cleavage was assessed. The cleaved CTD of RgpB was absent from *porQ* and both *porZ* mutants indicating that cleavage was blocked (Figs 2B and 3C).

### PorQ is required for OM-association of PorZ and surface location of secreted T9SS substrates

**Figure 4:**
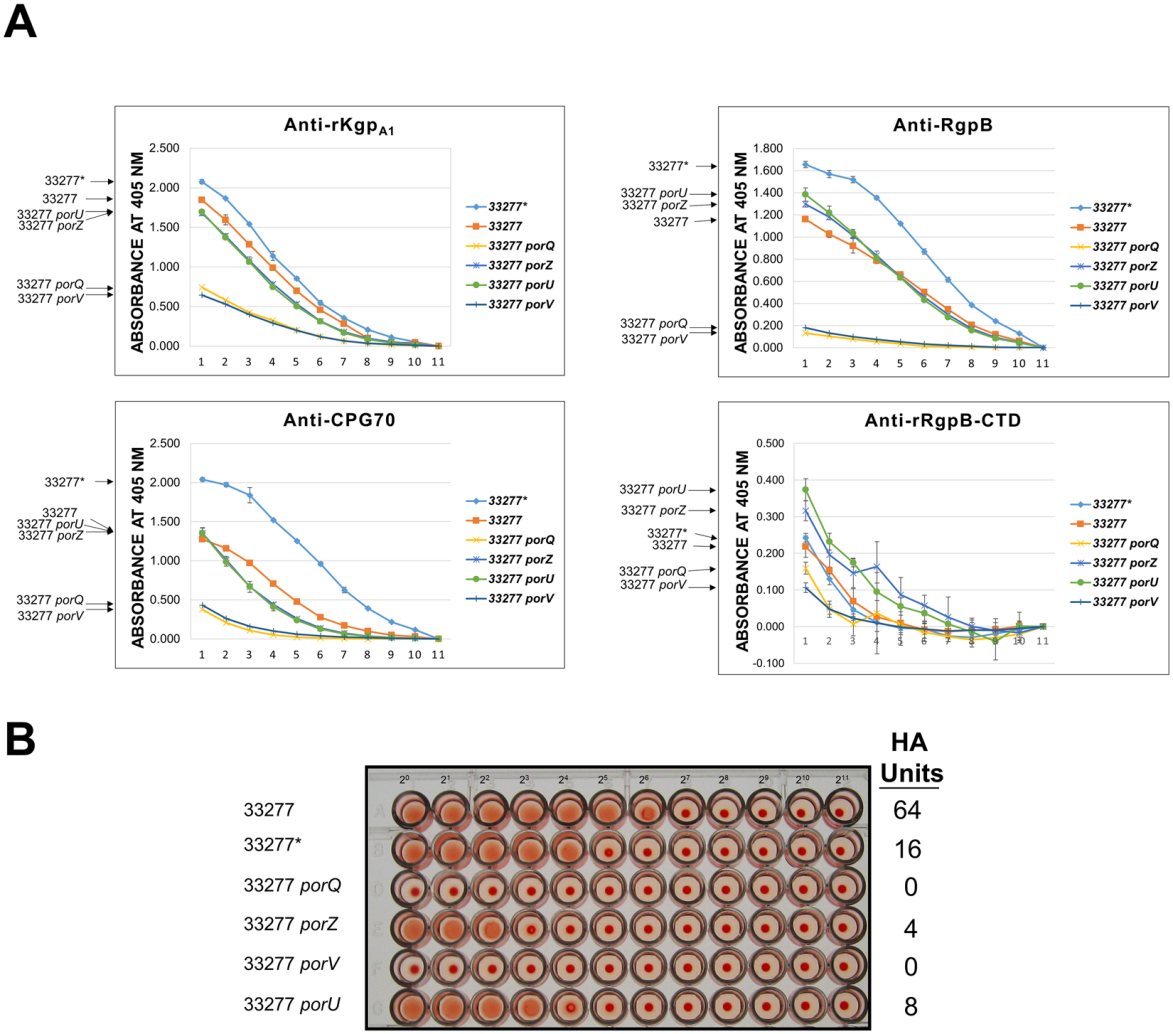
Secreted T9SS substrates are reduced but still present on the surface of the *PorZ* mutant. (**A**) Whole cell ELISAs. Starting antisera dilutions (dilution 1) for serial 2-fold dilutions for points 1–10 were 1/200 (anti-rRgpB CTD) or 1/400 (anti-RgpB, anti-rKgpA1 and anti-CPG70). Point 11 represents secondary anti-mouse IgG horse radish peroxidase conjugate only controls to which all values were normalised for each strain. Signal intensity order for strains are indicated on left hand side of figure. (**B**) Haemagglutination assays. Two fold serially diluted bacterial suspensions were mixed with an equal volume of 1% (v/v) erythrocyte suspension in U bottom microtitre plates and photographed after 3 h incubation at room temperature. Haemaggutination units were calculated per 50 µl of starting cells and are indicated on the right hand side.

Western blot of fractionated cell material from the *porQ* mutant showed that PorZ was substantially reduced in the membrane fraction and of increased relative abundance in the soluble fraction (Fig. 3A, anti-rPorZ) consistent with our finding that PorQ directly interacts with PorZ (Fig. 1). WC ELISA results showed that in general both *porV* and *porQ* mutants possessed the lowest abundance of proteins identified by four antisera raised against rKgp_A1_, CPG70, RgpB and rRgpB-CTD suggesting that little or none were present on the surface of these mutants (Fig. 4A). For the antisera to the three mature T9SS substrates, Kgp_A1_, CPG70 and RgpB, the general abundance order was WT strains > *porU*, *porZ* ≫ *porQ*, *porV*, suggesting that there was partial secretion of T9SS substrates to the surface in *porU* and *porZ* but none in *porQ* and *porV*. Anti-rRgpB-CTD, which detects surface RgpB with an uncleaved CTD, showed the highest level of RgpB-CTD in *porU* and *porZ* suggesting that uncleaved T9SS substrates were accumulating on the cell surface (Fig. 4A).

### Reduction of haemagglutination activity in attachment complex mutants correlates with level of surface CTD-proteins

Haemagglutination (HA) activity in *P. gingivalis* is associated with the T9SS substrates RgpA, Kgp and HagA^33, 34^ and would be expected to correlate with the surface level of these proteins. The HA activity of the *porU* and *porZ* mutants was reduced whereas the mutants lacking the integral OM proteins PorV and PorQ possessed no detectable HA activity which corresponded to a reduced or no surface presentation of T9SS substrates, respectively, in WC ELISA, (Fig. 4B).

### PorV binds directly to T9SS substrates in *porU* mutants

**Figure 5:**
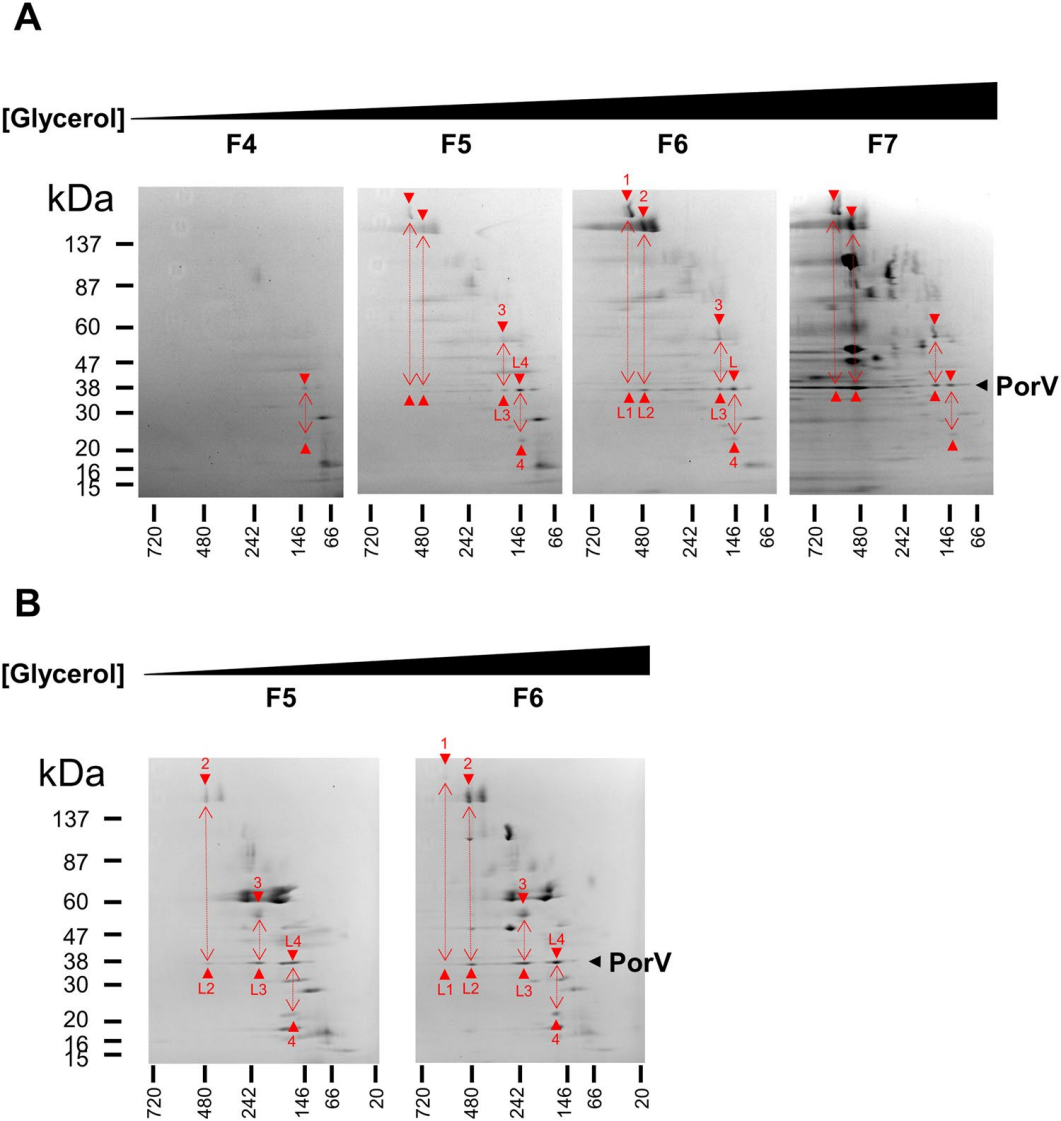
PorV interacts with immature T9SS substrates in *porU* mutants. Coomassie stained 2D BN-PAGE of rate-zonal glycerol density gradient centrifugation fractions of DDM solubilized OMVs from (**A**) W50 *porU* or (**B**) 33277 *porU*_*C690A*_. Fractions analysed are shown above. Red double sided arrows indicate proposed interacting proteins and red arrowheads point to individual protein spots, some of which were identified by MS and labeled for (**A**) as: L (L1, L2, L3, L4), PorV; 1, HagA; 2, mix of Kgp and RgpA; 3, TapA (PG2102/P59); 4, PG2172 (see Supplementary Table S3). Labeled for (**B**) as: L (L1, L2, L3, L4), PorV; 1, HagA (was not confirmed by MS); 2, mix of Kgp and RgpA; 3, RgpB; 4, PG2172 (see Supplementary Table S4).

The native proteins from OMVs of the W50 *porU* mutant were initially separated by 2D BN-PAGE directly, and although PorV appeared to have increased in native size relative to WT it was smeared (Supplementary Fig. S3) possibly due to a high content of OM lipids. The *porU* OMV lysate was therefore fractionated by glycerol density gradient centrifugation and fractions subjected to ultracentrifugation to pellet large complexes prior to 2D BN-PAGE (Fig. 5A). PorV could now be clearly observed to separate into distinct and different sized complexes. Each PorV complex co-aligned with a different protein identified as HagA (spot 1, 590 kDa), a mix of Kgp/RgpA (spot 2, 480 kDa), PG2102 (TapA, spot 3, 175 kDa) and PG2172 (spot 4, 140 kDa) (Fig. 5A and Supplementary Table S3). These proteins interacting with PorV were all T9SS substrates in their immature states based on size and the presence of CTD and pro domain peptides. The OMV lysate of the *porU* catalytic mutant, 33277 *porU*_*C690A﻿﻿*_, was also fractionated and PorV co-aligned with the T9SS substrates, HagA, a mix of Kgp and RgpA, PG2172 and RgpB (Fig. 5B and Supplementary Table S4). RgpB was aligned with PorV at a native size of 230 kDa and from its denatured size of 56 kDa, corresponded to pro-less form [IV] retaining its CTD.

### Evidence for PorZ binding to A-LPS

**Figure 6:**
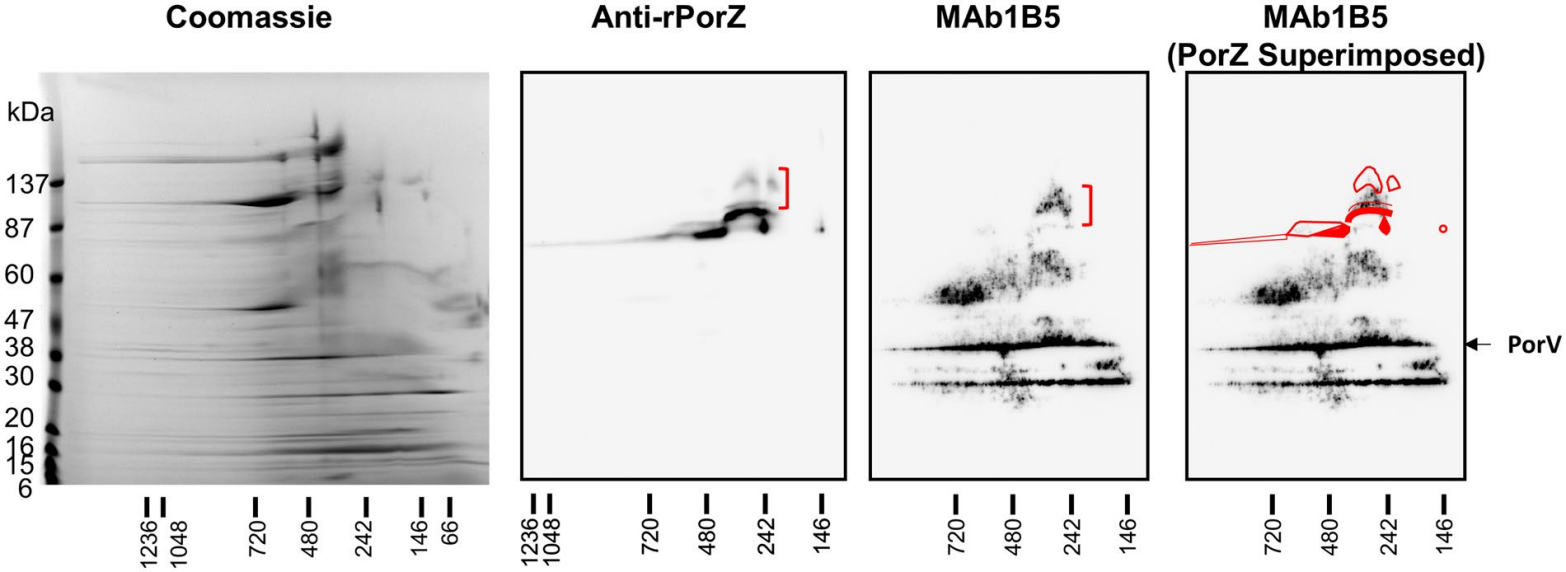
PorZ appears to bind A-LPS in the *porU* mutant. OMV lysate from the W50 *porU* mutant was subjected to 2D BN-PAGE and separately stained with Coomassie or sequential Western blots with anti-rPorZ then stripped and probed with anti-rPorV mouse antiserum followed by MAb1B5. The small population of PorZ co-aligning with MAb1B5 staining is indicated by red brackets.

It is possible that the role of PorZ may be to bind and present A-LPS to the PorU sortase. In the absence of PorU, PorZ might accumulate in an A-LPS-bound form. In 2D BN-PAGE of *porU*, PorZ was observed at its expected denatured MW of ~80 kDa, but in addition was observed at higher MWs of up to ~150 kDa suggesting interaction with other molecules (Fig. 6). A-LPS was mainly found at denatured MWs of approximately 30, 40, 50–70 and 90–130 kDa (Fig. 6, MAb1B5). The 90–130 kDa form of A-LPS and the high MW population of PorZ vertically co-aligned with each other and shared a similar triangular shape. The partial separation of PorZ and A-LPS in the 2^nd^ dimension, co-alignment and similar spot shape are all consistent with a non-covalent interaction that is partially resistant to denaturation in SDS.

### The *rgpB-CTDΔ6*^+^ mutant is partially defective in T9SS function

Our results demonstrate that in the absence of CTD cleavage by the PorU sortase, T9SS substrates accumulate in a PorV-bound form (Fig. 5). We therefore hypothesised that a T9SS substrate expressed with an uncleavable CTD would also accumulate in a PorV-bound form. To this end we constructed a strain expressing recombinant RgpB-CTDΔ6 containing a 6 amino acid deletion immediately C-terminal to the native CTD cleavage site^30^. In this cleavage mutant we predicted that the mutant RgpB-CTDΔ6 would bind and occupy PorV thereby partially excluding PorV from binding other CTD proteins and affecting their secretion. The *rgpB-CTDΔ6* gene was introduced into the interrupted *PG1795* locus in the deletion strain (*PG1795KO*) (Supplementary Fig. S4). The deletion of the PG1795 gene product had no effect on the T9SS as it had no effect on the level of pigmentation, on CTD-protein processing or the level of whole cell RgpA/B and Kgp activity (Supplementary Figs S5 and S6). The cleavage mutant, 33277 *PG1795KO::rgpB-CTDΔ6* ^+^, produced a reduced pigmentation of brown rather than black, had more cell surface gingipain activity (Supplementary Fig. S5), exhibited processing defects for only some CTD-proteins, particularly RgpB-CTDΔ6, and had an elevated level of free A-LPS (Supplementary Fig. S6). Together, the results indicate that the T9SS while perturbed in the cleavage mutant, was not greatly attenuated.

### Processing of RgpB by the T9SS is stalled in the *PG1795KO*::*rgpB-CTDΔ6*^+^cleavage mutant

**Figure 7:**
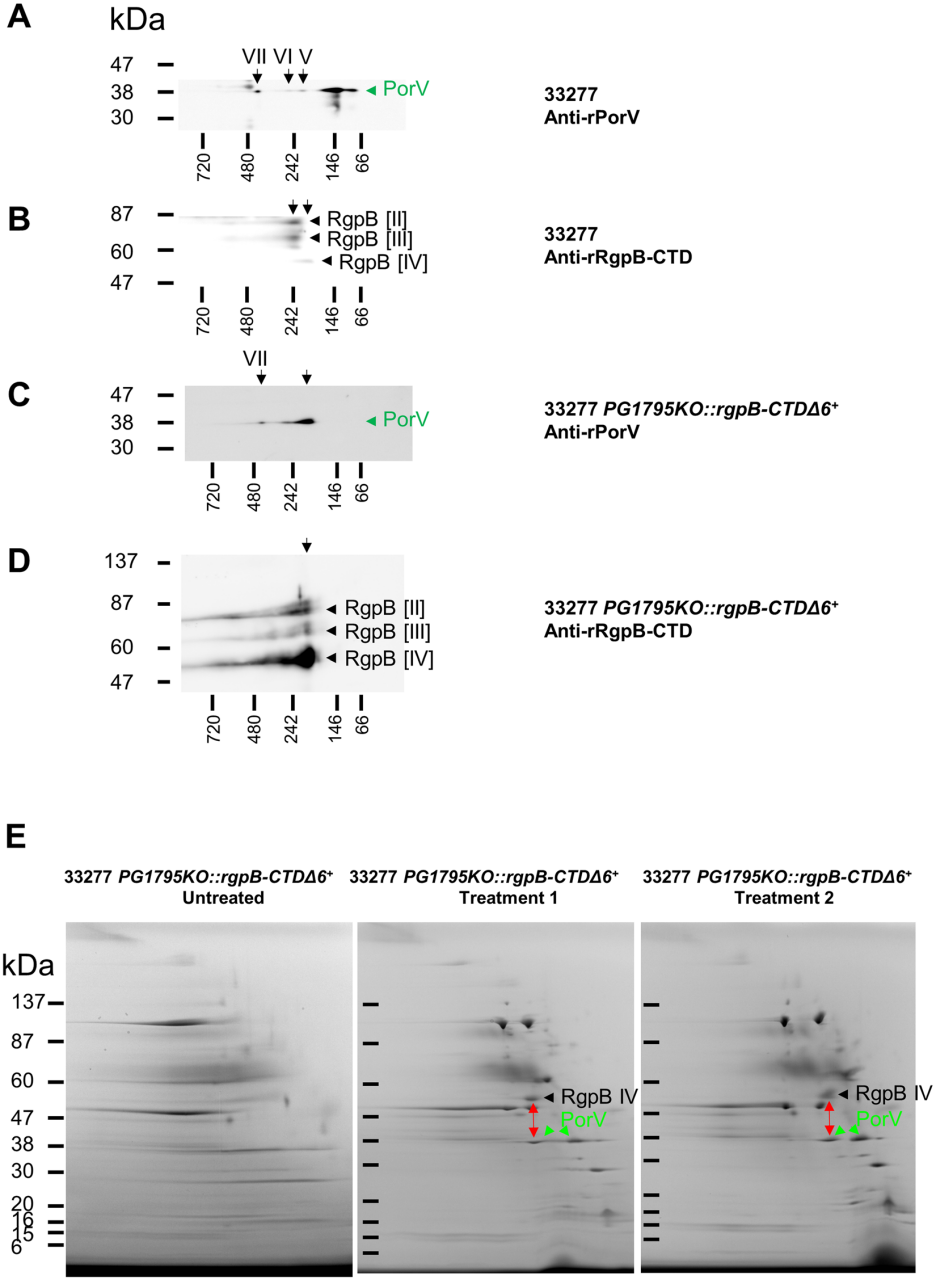
Processing of CTD cleavage mutant RgpB-CTDΔ6 appears to be stalled at an immature complex interacting with PorV. Western blot analyses of whole cell lysates from WT strain 33277 (**A** and **B**)^8^ and mutant strain 33277 *PG1795KO::rgpB-CTDΔ6* ^+^ (**C** and **D**) separation by 2D BN-PAGE and immunostained with anti-rPorV (**A** and **C**) and anti-rRgpB-CTD (**B** and **C**). (**E**) Coomassie stained 2D BN-PAGE analyses of OMV lysates from 3277 *PG1795KO::rgpB-CTDΔ6* ^+^ either untreated or washed by ultrafiltration (treatments 1 and 2).

RgpB processing in the CTD cleavage mutant, *PG1795KO::rgpB-CTDΔ6* ^+^, was assessed by 2D-BN-PAGE Western blot (Fig. 7). In whole cell lysate of the WT strain, the minor PorV spots in complexes labeled V and VI (Fig. 7A) are consistent with complexes that contained RgpB [II]:[III] and [IV], respectively, (Fig. 7B). However, the majority of PorV in WT was of low native size, approximately 150 kDa (Fig. 7A) and not interacting with any other protein (Supplementary Fig. S3A). In contrast, in the whole cell lysate of *PG1795KO::rgpB-CTDΔ6* ^+^, PorV existed in only two native complexes (Fig. 7C). The minor complex at 440 kDa was consistent with the attachment complex (Fig. 1A). The second more abundant PorV complex was a similar native size to PorV complex V in WT (Fig. 7A) and was found to co-align predominantly with RgpB [IV] (Fig. 7D). These results suggest that rRgpB-CTDΔ6 was unable to be cleaved and therefore accumulated in a PorV-bound form, ultimately binding to most of the PorV in cells.

The OMV lysates from *PG1795KO::rgpB-CTDΔ6* ^+^ and its parent, *PG1795KO*, were also analysed by 2D BN-PAGE and stained with Coomassie blue (Fig. 7E and Supplementary Fig. S7). Most of the proteins were smeared and unresolved for both strains. The OMV lysates were therefore washed by two different ultrafiltration procedures and re-analysed. The resolution of the OMV complexes for both strains was considerably better after both treatments (Fig. 7E and Supplementary Fig. S7). The major difference between strains was an abundant complex containing PorV and RgpB [IV] in the cleavage mutant (Supplementary Table S5). RgpB [IV] was primarily rRgpB-CTDΔ6 due to the detection of tryptic peptide fragments spanning the deletion and it confirmed that the CTD could not be cleaved causing the accumulation of RgpB [IV] in a PorV-bound state. Interestingly, the free form of PorV that does not interact with T9SS substrates was now observed in the OMV lysate of *PG1795KO::rgpB-CTDΔ6* ^+^ (Fig. 7E and Supplementary Table S5) but not in whole cells (Fig. 7C). The average molar ratio for rRgpB-CTDΔ6:PorV of treatments 1 and 2 (Supplementary Fig. S7) was 1.0:1.2 consistent with a 1:1 stoichiometry.

### Quantitative proteomics supports the role of PorV as a shuttle protein

**Figure 8:**
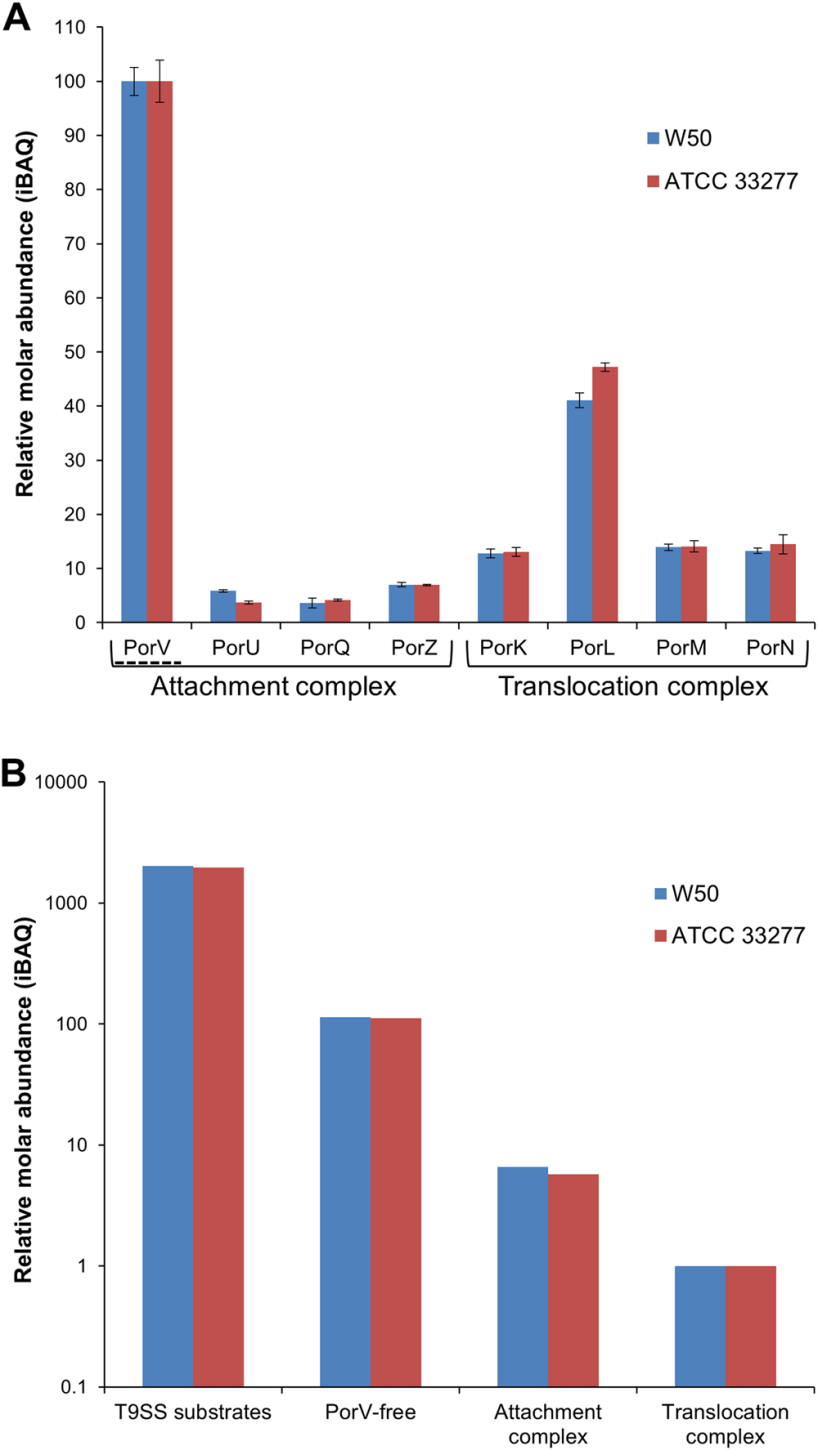
Stoichiometry of the proteins and complexes involved in substrate delivery. Whole cell lysates of WT strains W50 and ATCC 33277 were digested with trypsin and analysed by LC-MS/MS. Three replicate injections were analysed and quantitated using MaxQuant software. The iBAQ metric was used as an estimate of the relative molar abundance of each protein. (**A**) Relative abundance of components plotted on a linear scale with the abundance of PorV set to 100 for each strain. Error bars represent the standard deviation of the mean. PorV is mostly free with only a small portion present in the attachment complex. (**B**) Relative abundance of protein complexes plotted on a logarithmic scale. The translocation complex comprising PorK, PorL, PorM and PorN was set to a relative abundance of 1 based on the average iBAQ values of PorK, PorM and PorN. The iBAQ values of the other three categories were multiplied by 16 relative to the translocation complex to account for the differing number of subunits. The value of the attachment complex was taken as the average of the values for PorU, PorQ and PorZ. This value was then subtracted from the total PorV to give the value of free PorV.

The stoichiometry of the T9SS components was determined by quantitative label-free proteomics of whole cell lysates of WT strains. Over 1200 proteins were identified, but only the relevant data are shown (Supplementary Table S6). In both strains, PorV was in excess of other attachment complex components by approximately 16-fold while most translocation complex components were about twice as abundant as the attachment complex proteins, PorU, PorZ and PorQ (Fig. 8A). Interestingly, PorL was found to be three times more abundant than the other translocation complex components (Fig. 8A). Since the PorK/N complex is known to have at least 32 subunits per ring^27^, PorM may contribute a similar number of subunits to the overall complex while PorL may contribute approximately 96 subunits. The attachment complex, free PorV and PorV-RgpB were concluded to contain at most two subunits of each individual component based on native size and molar ratio. Thus, relative to the translocation complex, there was approximately a 6-fold excess of attachment complexes, a 110-fold excess of free PorV and a 2000-fold excess of T9SS substrates (Fig. 8B).

## Discussion

In our previous study, PorV was found to be essential for the secretion of T9SS substrates, and for the deacylation of lipid A^35^. We therefore concluded that PorV was associated with the deacylation of lipid A, and that it was crucial to allow the transport and conjugation of (deacylated) A-LPS to the secreted T9SS substrates. For this reason we named the protein LptO due to its association with the lipid A transport (Lpt) pathway. In this report we provide direct evidence to show that PorV is a *bona fide* multifunctional component of the T9SS as it not only binds and tethers the PorU sortase to the cell surface, but also binds to secreted T9SS substrates thus providing the link between OM translocated substrates and their presentation to the attachment apparatus. Additionally, we present evidence that the PorU sortase exists in an “attachment” complex with two new members, PorZ and PorQ, in addition to PorV.

2D BN-PAGE of WT OMVs fractionated by density gradient centrifugation reproducibly exhibited four co-aligned proteins, PorU, PorZ, PorV and PorQ, at a native size of 440 kDa (Fig. 1 and Supplementary Fig. S1). PorU and PorV were found in a subcomplex of lower size (270–330 kDa) either from a purified source^31^ or observed in a *rgpA rgpB kgp* mutant that could not synthesise A-LPS^8, 10^ demonstrating that PorU can bind directly to PorV. The association of PorZ and PorQ with PorU and PorV is a novel finding. In *porU*_*C690A*_, PorZ and PorQ were found to form a smaller subcomplex of 200 kDa suggesting that PorZ bound PorQ directly (Fig. 1). The absence of PorU and PorV from this complex explains the lower native size. Similarly, the absence of PorZ and PorQ from the PorU:PorV subcomplex explains its lower size, and together support the participation of all four proteins in the larger WT complex. Densitometry analyses of the various complexes are consistent with a simple 1:1 stoichiometry for the PorU-PorV and PorZ-PorQ subcomplexes, however analysis of the complete 440 kDa attachment complex suggested that PorV may be in excess. It is possible that the extra PorV subunit(s) may be required to stabilise the interaction between the two subcomplexes. The label-free MS quantitation of whole cell lysates was consistent with PorU, PorZ and PorQ being in 1:1:1 proportions, but could not confirm the excess of PorV in this complex since the bulk of PorV was free (Fig. 8). While the attachment complex was identified in OMVs, PorV was found in the same size complex (440 kDa) in whole cells by Western blot^8^ (Fig. 7) indicating that the attachment complex has the same composition in the cell membrane.

The findings of Lasica *et al*.^23^ show that PorZ and PorU are localised on the surface of the cell consistent with CTD cleavage and A-LPS conjugation being catalysed after secretion of T9SS substrates to the cell surface. They also found that mutation of *porU* does not affect the surface location of PorZ but conversely mutation of *porZ* blocks surface presentation of PorU. Our results show a direct interaction between PorZ and the PorQ outer membrane protein which provides an explanation for why PorZ can still be found on the surface in *porU*. This surface presentation of PorZ is prevented when half of its CTD is truncated^23^ which may indicate that this region is involved in the interaction with PorQ. The lack of surface PorU in *porZ* ^23^ may be explained by an unavailability of PorV due to preferential binding to abundant T9SS substrates excluding binding of the much lower abundant PorU.

The co-alignment and similar shaped immuno-stains of anti-rPorZ and MAb1B5 (Fig. 6) are consistent with the proposed interaction between PorZ and A-LPS and would provide the necessary function of recruiting A-LPS for the PorU sortase. The crystal structure of PorZ^23^ has revealed the presence of two seven bladed β-propeller domains a structure known to possess protein-protein and protein-substrate interaction properties leading to the authors’ proposal that PorZ may bind to sugars just like its nearest homologue, BT4663, a two component signal sensor binding to heparin and heparin sulphate in *Bacteroides thetaiotaomicron* ^23, 36^. In an A-LPS^-^ mutant, only the PorU:PorV subcomplex forms^8^. PorZ may need to bind to A-LPS in order for the PorZ:PorQ subcomplex to bind to PorU:PorV to form the WT attachment complex.

PorQ appears to anchor PorZ to the cell surface. Interestingly, *porQ* exhibited a more severely impaired secretion phenotype than *porZ*. In *porZ*, surface T9SS substrates were detected (Fig. 4A), there was some hemagglutination activity (Fig. 4B), and RgpB was processed to RgpB [IV] (Fig. 2). In contrast, secretion appeared completely blocked in *porQ* (Figs 3 and 4) suggesting that PorQ may have additional role(s) beyond anchoring PorZ.

While over 30 T9SS substrates have been identified in *P. gingivalis* ^6, 15, 37^, PorV was only found to interact with six T9SS substrates namely HagA, Kgp, RgpA, RgpB, TapA and PG2172 (Fig. 5). Besides PG2172, these tend to be the most abundant T9SS substrates which explains their preferential detection. Further PorV spots of lower intensity and definition were also observed, and presumably these represent interactions with additional T9SS substrates that were not clearly identifiable. The detection of PorV interacting with PG2172 given its low abundance^37^ is interesting and may imply a strong binding affinity.

The only known similarity amongst T9SS substrates is the presence of the CTD^6, 15^. Since the CTD can be detected on the cell surface in the *porU* mutant, it is likely that the CTD is the domain responsible for binding to PorV. Furthermore, since the *porV* mutant appeared to affect the secretion and modification of all T9SS substrates, we propose that all T9SS substrates that require PorU-processing will be delivered via PorV (see below). While it is known that the two most C-terminal residues of the RgpB CTD (VK) are essential for RgpB secretion and attachment^38^, it is uncertain whether these residues are involved in PorV binding or in an earlier step in the secretion pathway.

PorV has been found in three forms. In the WT, PorV is mostly found in the unloaded (free) form, but a small proportion is found in the attachment complex of 440 kDa comprising the attachment complex (Figs 1A and 7A)^8^. The third form represents the various complexes between PorV and individual T9SS substrates. In *porU*, many of these complexes can be observed, presumably since all substrates can be secreted and bound to PorV. We therefore designed the RgpB cleavage site mutant in order to limit the number of PorV complexes observed. As predicted, the expression of rRgpB-CTDΔ6 with an uncleavable CTD resulted in the specific accumulation of the PorV:rRgpB-CTDΔ6 complex (Fig. 7). In whole cell lysates (Fig. 7C,D), PorV was almost entirely found in complex with rRgpB-CTDΔ6, with no observable presence of the unloaded form. This means that T9SS substrates that have emerged from the secretion pore would have no free PorV to bind to, and therefore would accumulate in the periplasm, just as they do in the *porV* mutant^35^. With the synthesis of each new PorV protein, native substrates would be processed normally until the new PorV bound irreversibly to the next rRgpB-CTDΔ6.

Interestingly, the RgpB-CTDΔ6 cleavage site mutant also produced a PorV spot at 440 kDa (Fig. 7C) consistent with the presence of the complete attachment complex. Unlike the whole cell lysates, the OMV samples of the RgpB-CTDΔ6 cleavage site mutant contained an appreciable amount of the unloaded form of PorV (Fig. 7E). Since OMVs appear to lack some of the essential components of the T9SS such as PorK, PorM, PorL, PorW and Sov^37^ it is likely that translocation of T9SS substrates present in the lumen of the OMVs is not possible. Therefore, the presence of unloaded PorV in the OMVs suggests that either the rRgpB CTDΔ6:PorV complex slowly dissociates in OMVs and cannot be replenished by further translocation of substrates across the vesicle membrane or that PorV loaded with other CTD-proteins with cleavable CTDs are processed further in the OMVs. It is also possible that rRgpB-CTDΔ6 can be slowly processed by PorU allowing the observed recycling of PorV in OMVs.

**Figure 9:**
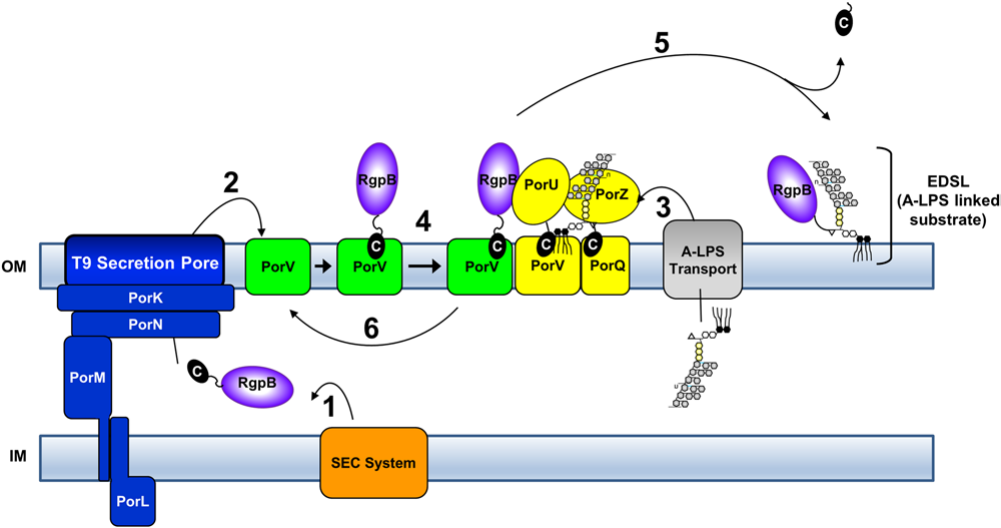
Model of Type IX Secretion. T9SS substrates have a conserved C-terminal domain (CTD) that directs their secretion across the outer membrane (OM). Substrates such as RgpB and also the T9SS components PorU and PorZ are transported across the inner membrane (IM) via the SEC translocon via their N-terminal leader sequence (Step 1). They are then secreted across the OM via the T9SS translocation apparatus that we propose includes PorK, PorL, PorM, PorN and possibly others, and secreted proteins are held on the cell surface through interaction with PorV (Step 2). The A-LPS anchor is independently transported to the outer leaflet of the OM (Step 3) and is recruited by the attachment complex consisting of PorV, PorU, PorQ and PorZ. PorV delivers the substrates to the PorU sortase (Step 4) for CTD cleavage and conjugation to the A-LPS anchor to produce the EDSL (Step 5). All members of the attachment complex are essential for Step 5 to occur. The cleaved CTD is then released to allow recycling of PorV (Step 6).

Figure 9 shows an updated model of the T9SS. Substrates are proposed to be directed to the secretion pore via a secretion signal located in their CTDs and then passed to PorV. Binding to PorV is presumably also via the CTD. The accumulation of T9SS substrates in the periplasm of the *porV* mutant^35^ and the absence of cell surface substrates^7^ indicates that the secretion pore does not secrete substrates unless PorV is present. In turn, this suggests that PorV may transiently bind to the secretion pore complex before collecting substrates. Since substrate-loaded PorV accumulates in strains unable to cleave the CTD (*porU* and *PG1795KO::rgpB-CTDΔ6*), it is likely that once PorV is loaded with substrate it is able to deliver them to the attachment complex for CTD cleavage and conjugation to A-LPS. The best chance of observing this intermediate substrate loaded attachment complex would be in a strain that accumulated just a single substrate-PorV complex such as *PG1795KO::rgpB-CTDΔ6*. However, while this mutant showed a strong PorV spot at 440 kDa corresponding to the attachment complex, there was no PorV spot observed at a higher native mass corresponding to a substrate loaded attachment complex. Therefore, it may be that the delivery of substrates to PorU only requires a transient interaction. Consequently, we propose that PorV functions as an outer membrane shuttle protein collecting substrates from the secretion pore and delivering them to the PorU sortase.

To our knowledge, this is the first report of an outer membrane protein functioning as a shuttle protein. Since free PorV is in considerable excess over both secretion channels and attachment complexes, it is likely that PorV delivers its cargo by diffusion along the OM. The finding that OMVs contain an abundance of PorV as well as the other members of the attachment complex, but not the members of the secretion apparatus^37^ would also seem to favour a diffusion model since it implies that free PorV and the members of the attachment complex are not permanently associated with the secretion channels *in vivo*. In Gram negative bacteria, OM fluidity studies have shown that Omps are not uniformly distributed in the OM but are found concentrated in specific areas or “islands”^39^. This together with photobleaching recovery experiments has led to the understanding that the OM is relatively static (not fluid) but permitting local diffusion within the timeframe observed (<1 min to 15 min)^39^. The abundance of outer membrane proteins in the “islands” are thought to limit the diffusion rate of neighbouring proteins. If *P. gingivalis* behaves similarly to these other species, it would be expected that newly synthesized T9SS pores together with the outer membrane proteins PorV and PorQ would be inserted into the OM localized at an island. Since PorU and PorZ are secreted by the T9SS pore they would remain in close proximity even after being captured by PorV and PorQ to form attachment complexes. Then when newly secreted CTD proteins are captured by PorV, diffusion across only a short distance is required to deliver the cargo to an attachment complex to facilitate covalent linkage to A-LPS. Since T9SS substrates are anchored to the OM by a relatively small molecule (lipid A), they may be able to diffuse more readily than the outer membrane proteins allowing them to spread over the entire surface of the cell to form the EDSL.

## Materials and Methods

### Bacterial strains and culture conditions

*Porphyromonas gingivalis* WT strains W50 (ATCC 53978) and ATCC 33277 were from the Oral Health Cooperative Research Centre, University of Melbourne, Australia. Isogenic mutants of these WT strains were W50 *porU* (W50 *PG26*), 33277 *porU*_*C690A*_ (33277 *PG26C690A*)^7^ and 33277 *porV* ^35^. Construction of *porZ* mutants (W50 *porZ*, 33277 *porZ*), *PG1795* deletion mutant (33277 *PG1795KO*) and RgpB cleavage mutant expressing recombinant rRgpB-CTDΔ6 (33277 *PG1795::rgpB-CTDΔ6* ^+^) are described in the Supplementary Materials and Methods. ﻿Bacterial strains are listed in Supplementary Table S7.  ﻿ The ATCC 33277* and 33277 *porQ* mutant have been previously described^11^ and were a kind gift from Professor Koji Nakayama. *P. gingivalis* strains were grown on TSBHI blood agar or TSBHI broth as previously described^7, 35^ with appropriate antibiotic except for the final passage. For plasmid constructions, competent α–select Gold *Escherichia coli* (Bioline) and BL21-CodonPlus (DE3)-RIPL (Stratagene) strains were grown on Luria-Bertani (LB) medium.

### DNA Isolation PCR and PCR purification

Plasmid DNA was purified using QIAprep Spin Miniprep Kit (Qiagen). Genomic DNA was obtained according to Chen and Kuo^40^. Primers used in this study are listed in Supplementary Table S8. PCR reactions used for cloning were amplified using 0.02 units/µl of Q5 Hot Start High Fidelity DNA polymerase (New England Biolabs) in 1 X buffer supplied by the manufacturer, 0.2 mM dNTP, 0.3 µM each of forward and reverse primers.

### Fractionation of *P. gingivalis* cultures

Whole cell lysates, membrane and soluble fractions, and filtered and clarified culture fluid (CCFF) for SDS PAGE and Western blots were prepared as previously described^7^.

Whole cell lysates for BN PAGE analysis were obtained from log phase cells grown in TSBHI broth medium, washed in PBS and solubilised in BN PAGE lysis buffer containing 1% (w/v) n-dodecyl-β-D-maltoside and proteinase inhibitors^8^.

OMVs were isolated from strains grown to late log phase and solubilized in BN PAGE lysis buffer at a wet weight between 90–300 mg/ml as previously described^8^. If culture fluid was not prefiltered before pelleting OMVs then it was additionally centrifuged 12000 *g* for 20 min at 4 °C.

Rate-zonal glycerol density gradient centrifugation of OMV lysates was performed as previously described^8^. Large scale glycerol density gradient fractionation of the OMV lysate was obtained by centrifugation of culture at 8000 *g* for 20 min at 4 C, supernatant was spun again at 12000 *g* for 20 min at 4 °C and then OMVs pelleted at 170000 *g* for 45 min at 8 °C. The OMV pellets were resuspended in 1 x NativePAGE^TM^ buffer (Life Technologies) containing 1% (w/v) n-dodecyl-β-D-maltoside (DDM), 20 mM TLCK and 10 µl/ml PIC (Sigma-Aldrich) as previously described^8^. Cleared lysate (3 ml) was applied to a 9 ml step glycerol gradient comprising of equal volumes of 70%, 60%, 40%, 33% (w/v) glycerol buffer^8^ and centrifuged at 40,000 rpm (SW40 Ti rotor, Beckman) for 46 h, 25 min at 8 °C then 0.5 ml fractions collected.

### 2D BN-PAGE

The 2D BN-PAGE protocol has been previously described^8^.

### SDS-PAGE and Western blot

Fractionated samples were separated by reducing SDS-PAGE and proteins transferred to nitrocellulose membranes. Transferred proteins were visualized by staining with Ponceau S and destained in PBS containing 0.05% v/v Tween 20 (PBST). Membranes were blocked for 1 h at room temperature with 5% w/v nonfat skim milk powder in PBST for polyclonal sera or with 1% w/v bovine serum albumin in PBST for monoclonal antibody. The blocked membranes were incubated with the appropriate primary mouse antibody followed by goat anti-mouse IgG conjugated to horse-radish peroxidase and detected by chemilluminescence as described previously diluted in respective blocking solution^35^. Anti-rPorZ was raised in mice to the N-terminal half of mature PorZ (Q^24^-D^400^) expressed from pPG1604N (see Supplementary Materials and Methods) in *E. coli* cells BL21-CodonPlus (DE3)-RIPL. Expressed His-tagged rPorZ was purified using PrepEase His-tagged protein purification mini kit-High Yield (usb) followed by ion exchange chromatography using a Mono Q 5/50 GL column (GE Healthcare) and 20 mM Bis-Tris-HCl, pH 7, with a 30 ml linear gradient of 0–0.75 M NaCl and injected into mice for raising antiserum as previously described^35^. Anti-rPorV (anti-rLptO), anti-rRgpB-CTD (anti-rRgpB-CTD(422)) and anti-rKgp_A1_ were raised against recombinant His-tagged proteins^7, 35, 41^. Native RgpB was purified as for native CPG70 to raise specific mouse antisera^42^. Mouse monoclonal antibody 1B5 (MAb1B5) that recognizes the epitope Manα1–2Manα1-phosphate found on A-LPS^43^ was a gift from Professor M. A. Curtis.

### Ultrafiltration treatment of OMV lysates

OMV lysates were washed by ultrafiltration using Ultracel-100K with a nominal molecular weight limit of 100,000 Da that concentrates proteins without excessive concentration of the detergent. Treatment 1 diluted the OMV lysate in BN PAGE sample buffer containing 0.5% w/v DDM then the sample was concentrated to the original volume. Treatment 2 included treatment 1 with an additional wash step using dilution in BN PAGE sample buffer containing 0.1% w/v DDM.

### Whole cell ELISA

Cells were grown on TSBHI agar without blood (TSBHI*) with appropriate antibiotic and then passaged once on TSBHI* without antibiotics. After 4 days growth cells were scraped off, washed twice in TC150 buffer (50 mM Tris, pH 7.4, 150 mM NaCl and 5 mM CaCl_2_), resuspended in formalin saline (0.5% v/v formaldehyde in 8.5 g/l NaCl) and prepared for whole cell ELISA as previously described^7^.

### Haemagglutination assay

Haemagglutination (HA) assays were performed as described previously^35^. HA units were calculated per 50 µl of undiluted sample from the reciprocal of the dilution that produced the endpoint of haemagglutination considered to be 1 HA unit.

### In-gel digestion and peptide extraction

Protein spots or bands were excised with a uni-core 1.20 mm diameter circular punch (GE Healthcare Life Sciences) or scalpel blade. Gel pieces were washed, reduced, alkylated and digested with trypsin as described previously^35^. For simple protein identification, the digested peptides were acidified with trifluoroacetic acid (TFA) prior to MS analysis. For the quantitative analysis, the acidified digests were removed from the gel pieces into other tubes, and the gel pieces sequentially extracted with (i) 0.1% TFA, (ii) CH3CN/0.1%TFA (1:5), and (iii) CH3CN/0.1% TFA (1:1) and combined with the original digest solutions. The combined extracts were concentrated in a vacuum centrifuge prior to MS.

### MALDI-TOF MS

For simple protein identification of isolated 2D BN-PAGE spots, MALDI-TOF analysis was usually sufficient for unambiguous protein identification. Samples were applied to a 600 um anchorchip plate using the “thinlayer” technique, and spectra acquired using an Ultraflex III MALDI TOF/TOF instrument (Bruker, Bremen, Germany) in positive reflectron mode as described previously^44^. Peptide mass fingerprint (PMF) searches were conducted against the *P. gingivalis* W83 or *P. gingivalis* ATCC 33277 protein sequence databases for W50 and ATCC 33277 strains respectively using the Mascot v2.2 search engine (Matrix Science, London, UK). The following search settings were employed: Enzyme = trypsin, mass tolerance = 50 ppm, missed cleavages = 0 or 1, fixed modifications = carbamidomethyl (Cys), optional modifications = oxidation (M). Proteins were considered identified when the Mascot score was at least 46 (p < 0.05). The protein matches were validated by multiple replicates and by the close match of measured MW (from SDS-PAGE dimension) and the expected MW of the protein.

### LC-MS/MS

LC-MS/MS analysis was using either a capillary HPLC (Ultimate 3000, Thermo) in series with an ion trap MS (Esquire HCT Ultra, Bruker)^35^ or an Ultimate 3000 nanoLC in series with a Q-Exactive Plus Orbitrap MS (Thermo)^45^. Search settings for the ion trap data were: Enzyme = trypsin, MS tolerance = 1.5 Da, MS/MS tolerance = 0.8 Da, missed cleavages = 1, fixed modifications = carbamidomethyl (Cys), optional modifications = oxidation (M). For the orbitrap data, the MS tolerance was set at 10 ppm and the MS/MS tolerance at 0.2 Da. Proteins were considered identified when at least two peptides were identified that were significant (p < 0.05). Peptides identified with a Mascot score of <15 were excluded from the identified peptide count. The quantitative analysis of whole cell lysates was conducted as above on an Ultimate 3000 nanoLC in series with an Orbitrap Fusion Lumos Tribrid MS (Thermo) with a 90 min run time^46^. Three replicate injections of both the ATCC 33277 and W50 whole cell lysates  were analyzed.

### Quantitation of 2D-gel spots by ImageQuant software

Quantitation of vertically aligned protein spots separated by 2D BN-PAGE was performed using ImageQuant software (GE Healthcare)^8^.

### Quantitation of whole cell lysates by MaxQuant

Raw MS files were analysed by MaxQuant (Ver 1.5.3.30) using label-free quantification^47^. The default parameters were used for LFQ except that quantification with 1 peptide was allowed, match between runs was allowed. The iBAQ metric was utilised as it more closely reflects molar abundance. The mean and standard deviation iBAQ values were calculated from the three replicate injections.

### Data Availability Statement

All data generated or analysed during this study are included in this published article (and its Supplementary Information files).

## Electronic supplementary material

Supplementary Information
