# Supplementary material for: PorV is an Outer Membrane Shuttle Protein for the Type IX Secretion System
Source: Sci Rep. 2017 Aug 18;7:8790. doi: 10.1038/s41598-017-09412-w (PMC5562754; doi:10.1038/s41598-017-09412-w)
Supplement: Supplementary file 1 — Supplementary Information [file 41598_2017_9412_MOESM1_ESM.pdf]

PorV is an Outer Membrane Shuttle Protein for the Type IX Secretion System

Michelle D. Glew, Paul D. Veith, Dina Chen, Dhana G. Gorasia, Ben Peng and Eric C. Reynolds*

Supplementary figures

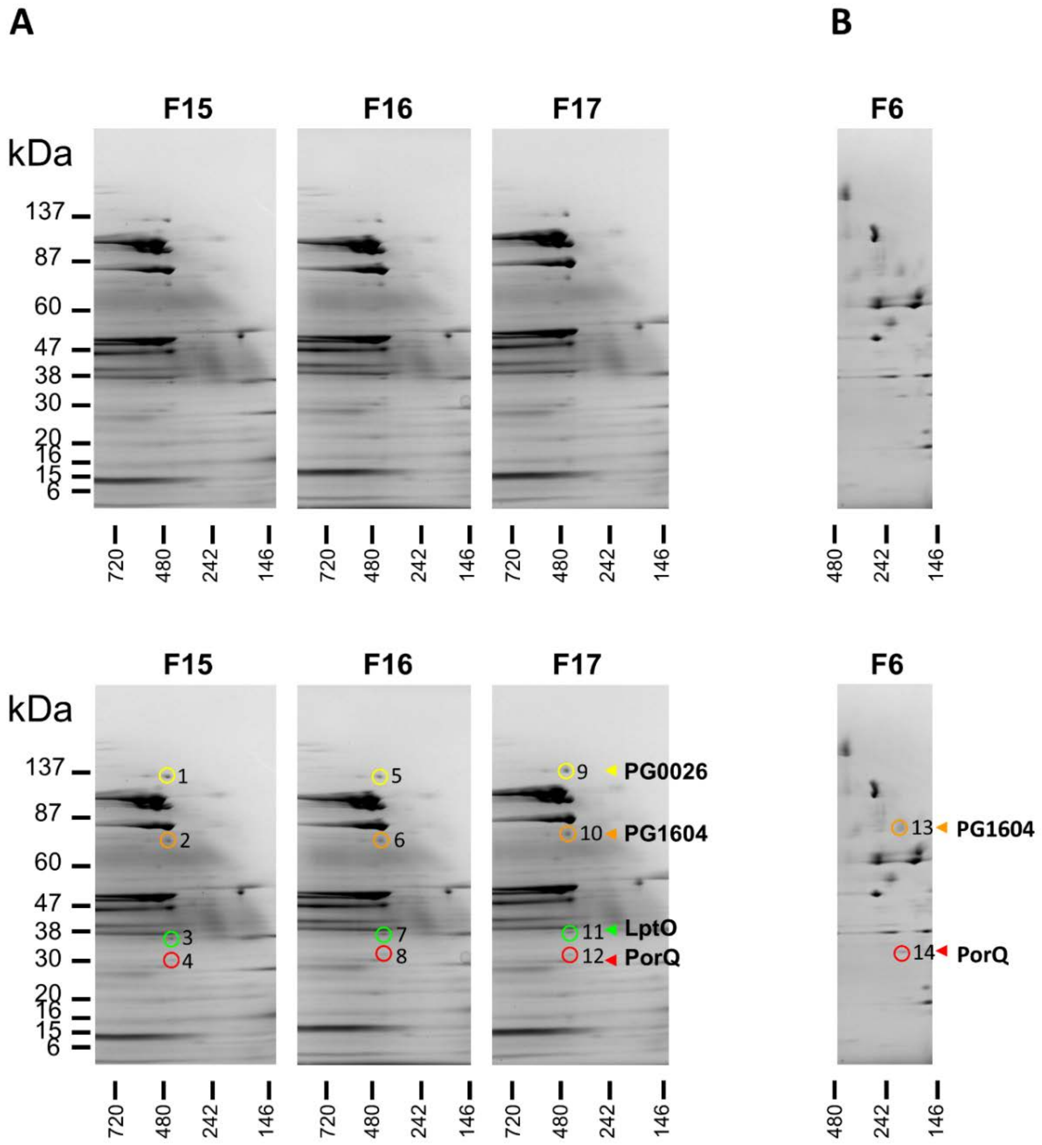

Supplementary Fig. S1. 2D BN-PAGE analysis of OMV lysates for MS identification. Coomassie stained 2D BN-PAGE analyses for OMV lysates from (**A**) large scale glycerol gradient fractions (F15, F16, F17) of crude W50 OMV lysate, (**B**) small scale glycerol gradient fraction F6 of W50 *porU*$_{C690A}$. Duplicate gels are shown below with circled and numbered protein spots taken for MS analysis (see Supplementary Table S1). NativeMark protein standard (Life Technologies) was used for first dimension. Second dimension protein standard was pre-stained BenchMark Ladder (Life Technologies) calibrated to BenchMark Protein Ladder (Life Technologies).

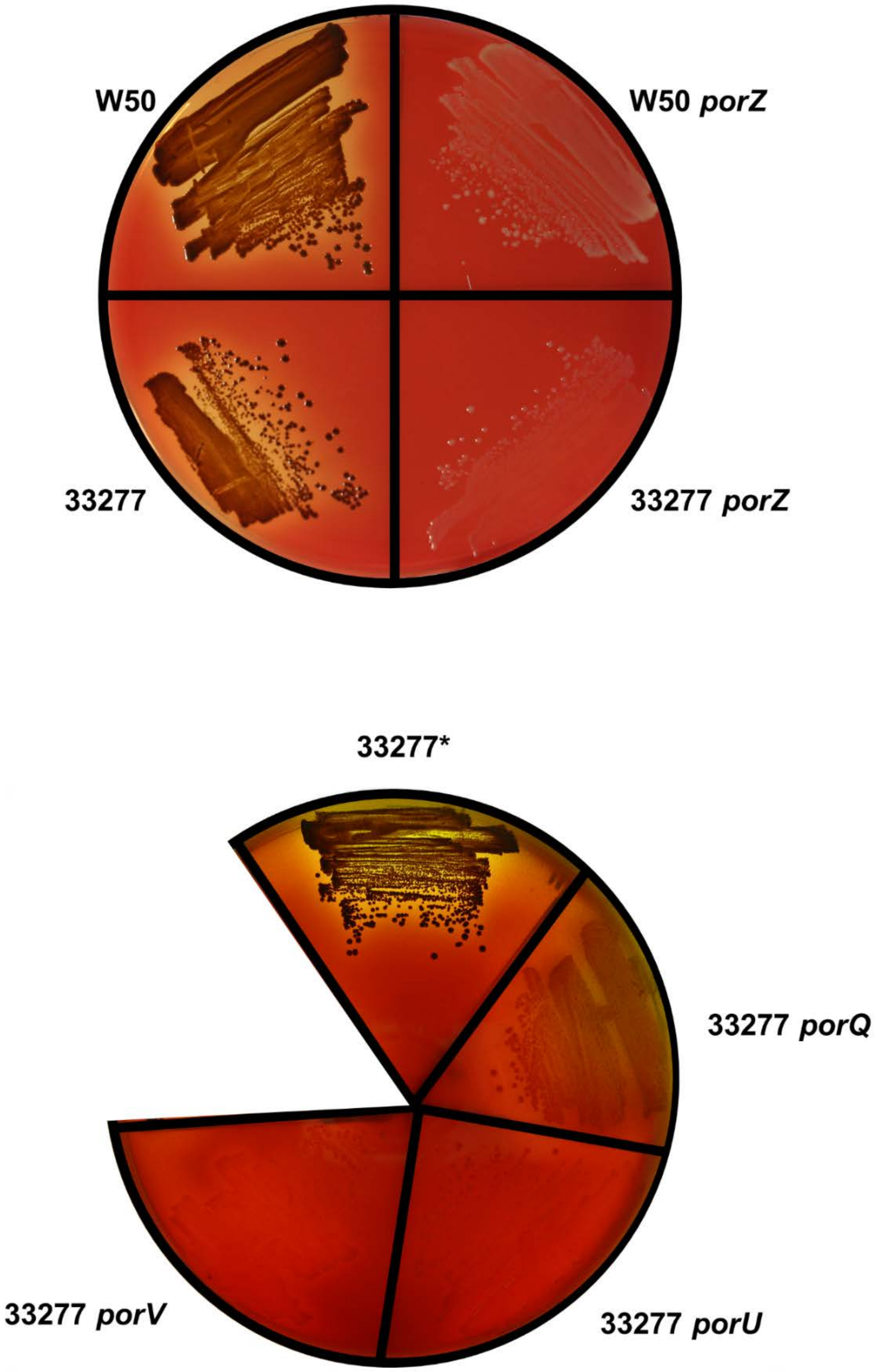

Supplementary Fig. S2. The *porQ* and *porZ* mutants do not produce pigmentation. *P. gingivalis* strains were grown anaerobically on TSBHI agar medium for 7 days at 37°C.

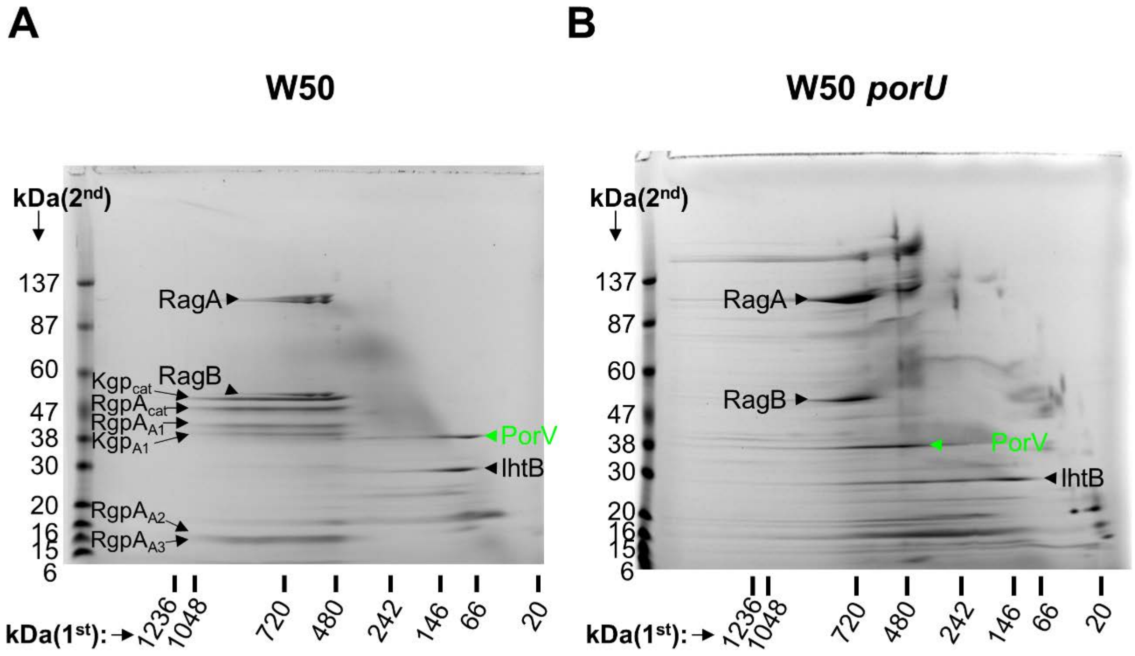

Supplementary Fig. S3. PorV native size change in *porU* mutant. Coomassie stained 2D BN-PAGE-gel for OMV lysates from W50 (**A**) (Glew et al. 2014) and W50 *porU*. Green arrowhead points to location of PorV.

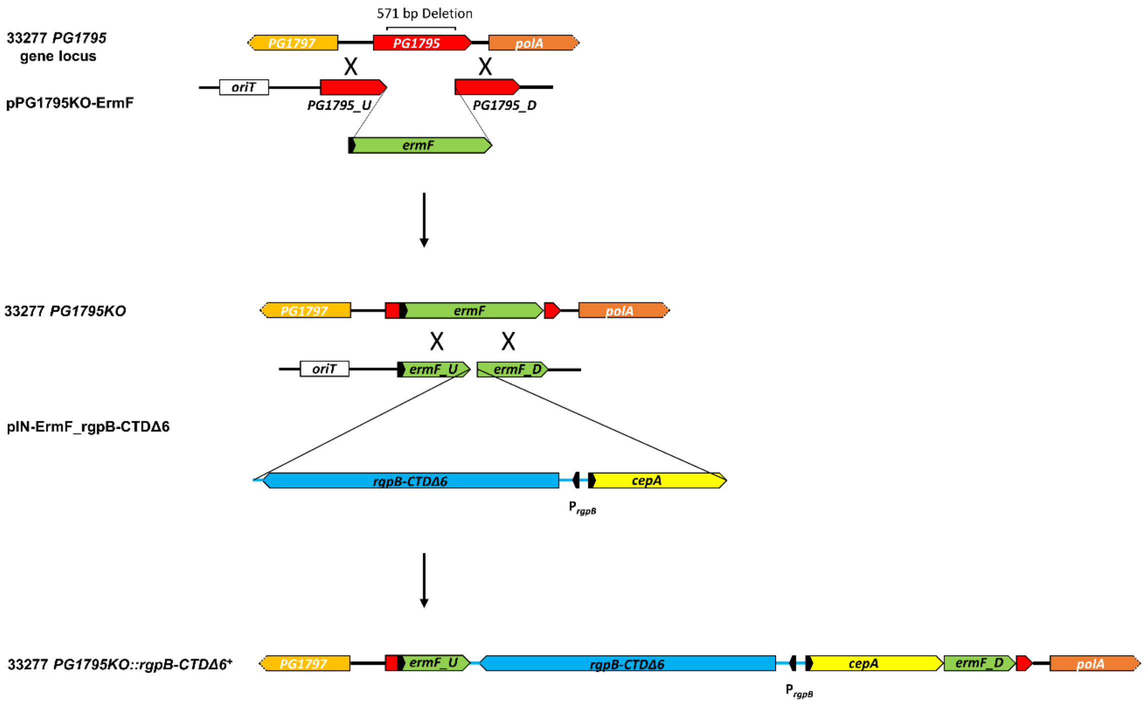

Supplementary Fig. S4. Schematic representation of strain loci and plasmids used for homologous recombination for the construction of strains 33277 *PG1795KO* and 33277 *PG1795KO::rgpB-CTDΔ6*⁺. Arrowed boxes are gene open reading frames shown to scale except where only the partial gene is shown (stippled arrow ended box). Two crosses indicate the homologous regions that undergo homologous recombination between the parental strain locus and the plasmid to create the mutant strain.

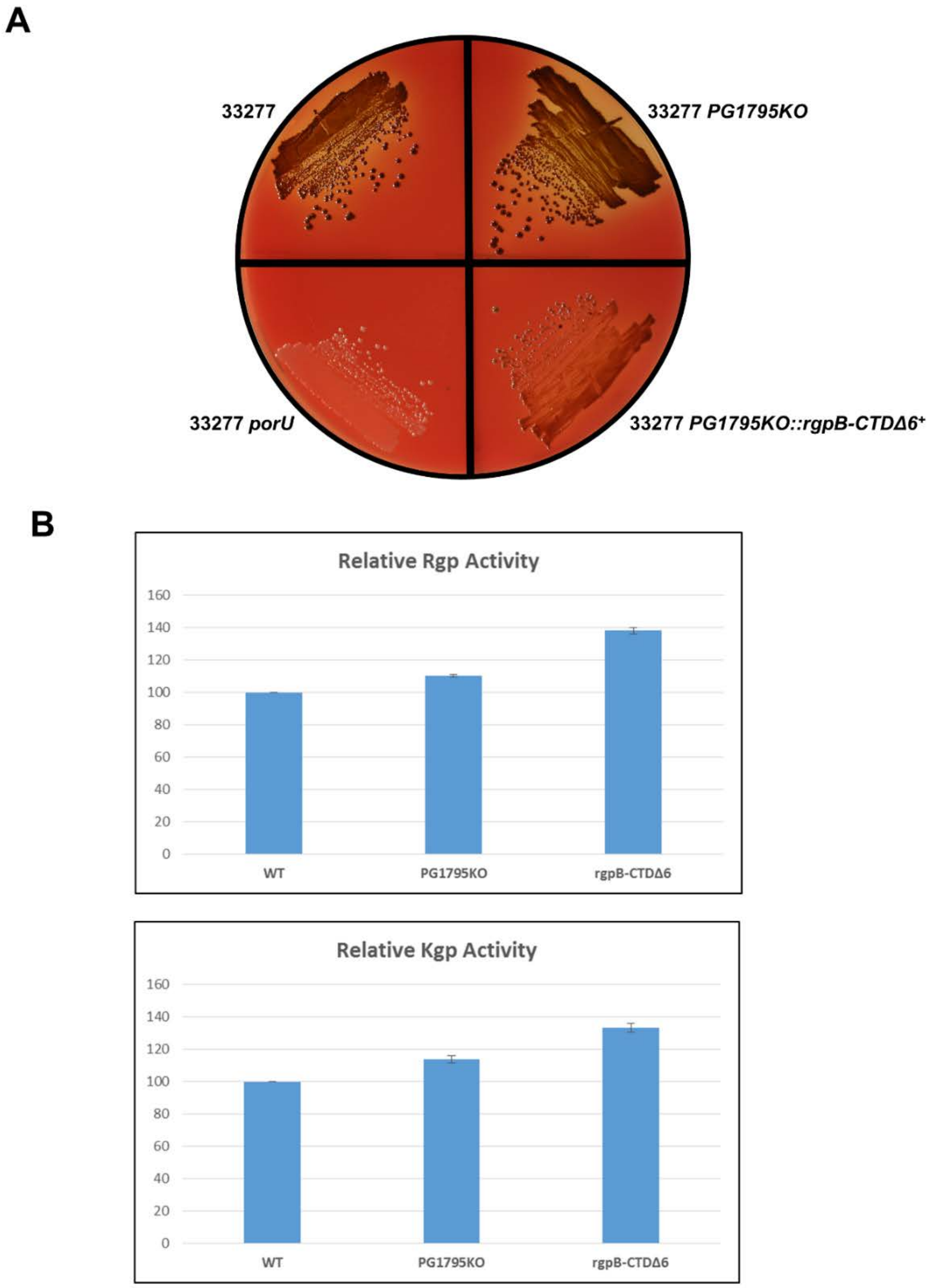

Supplementary Fig. S5. Pigmentation and gingipain activities. (**A**) Pigmentation of strains after growth on blood agar medium. (**B**) ATCC 33277 (WT) whole cell Rgp and Kgp activities were used as references for whole cell Rgp and Kgp relative activities, respectively, for strains 33277 *PG1795KO* and 33277 *PG1795KO::rgpB-CTDΔ6*$^{+}$ (rgpB-CTDΔ6).

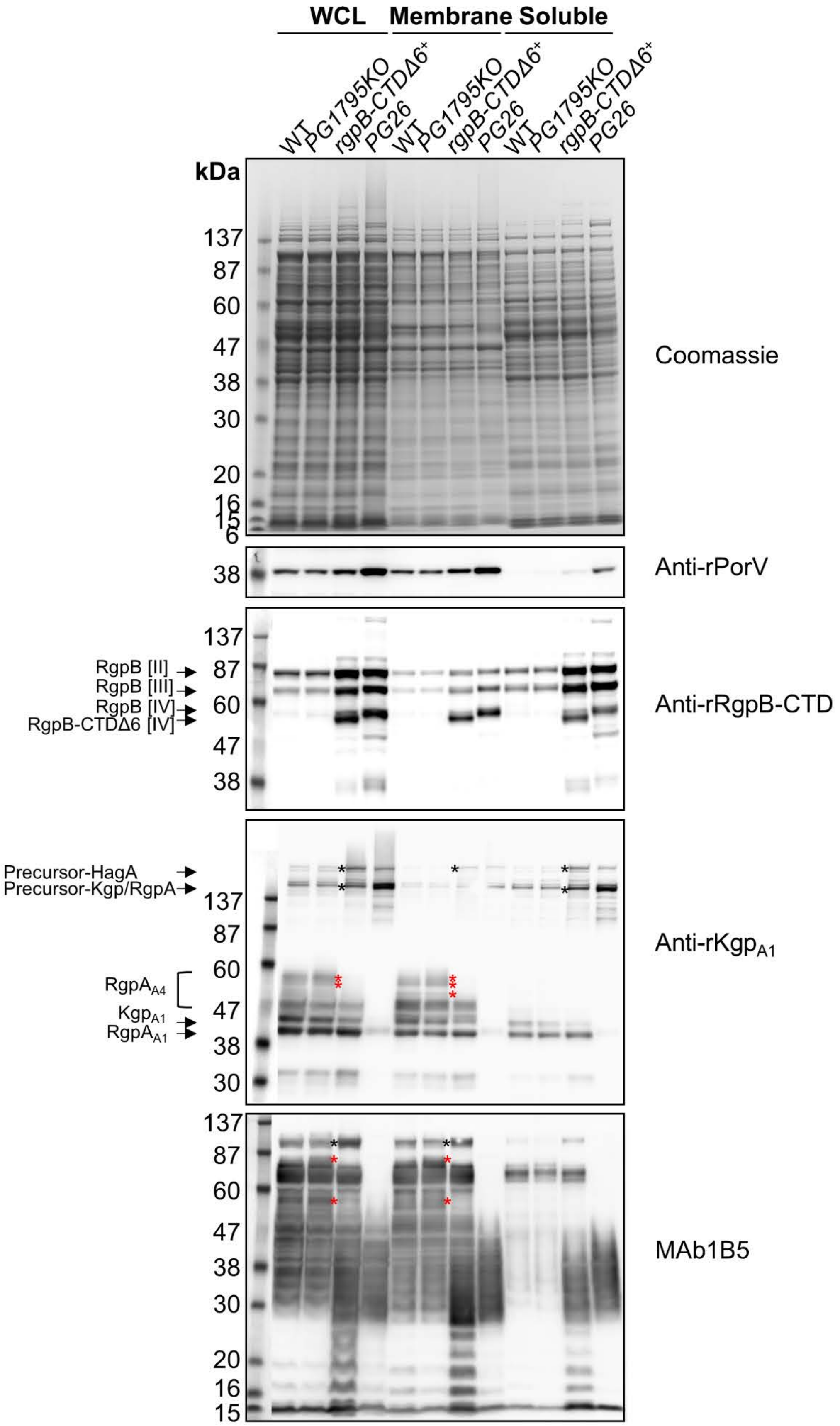

WCL
Membrane
Soluble
WT
PG1795KO
rgpB-CTDΔ6+
PG26
kDa
137
87
60
47
38
30
20
16
15
6
Coomassie
Anti-rPorV
RgpB [II]
RgpB [III]
RgpB [IV]
RgpB-CTDΔ6 [IV]
Anti-rRgpB-CTD
Precursor-HagA
Precursor-Kgp/RgpA
RgpA A4
Kgp A1
RgpA A1
Anti-rKgp A1
MAb1B5

Supplementary Fig. S6. Western blot analyses of quantitively fractionated *P. gingivalis* cultures for cleavage mutant and related strains. Proteins from strains ATCC 33277 (WT), 33277 *PG1795KO* and 33277 *PG1795KO::rgpB-CTDΔ6*$^{+}$ were grown to the same optical density (OD650nm of 0.8). SDS-PAGE loading of fractionated cells was normalized to 50 µL of culture and subjected to reducing SDS-PAGE using 10% Bis-Tris gels (MOPS running buffer) and proteins transferred to nitrocellulose membranes. The immunoblots were probed with anti-rPorV (diluted 1:500), anti-rRgpB-CTD (diluted 1:1000), anti-KgpA1 (diluted 1:1000) and MAb1B5 (diluted 1:100). S, protein standard, pre-stained BenchMark Protein Ladder calibrated to BenchMark Protein Ladder. Red asterisks indicate missing bands in cleavage mutant relative to WT. Black asterisks indicate bands with increased signal level relative to WT.

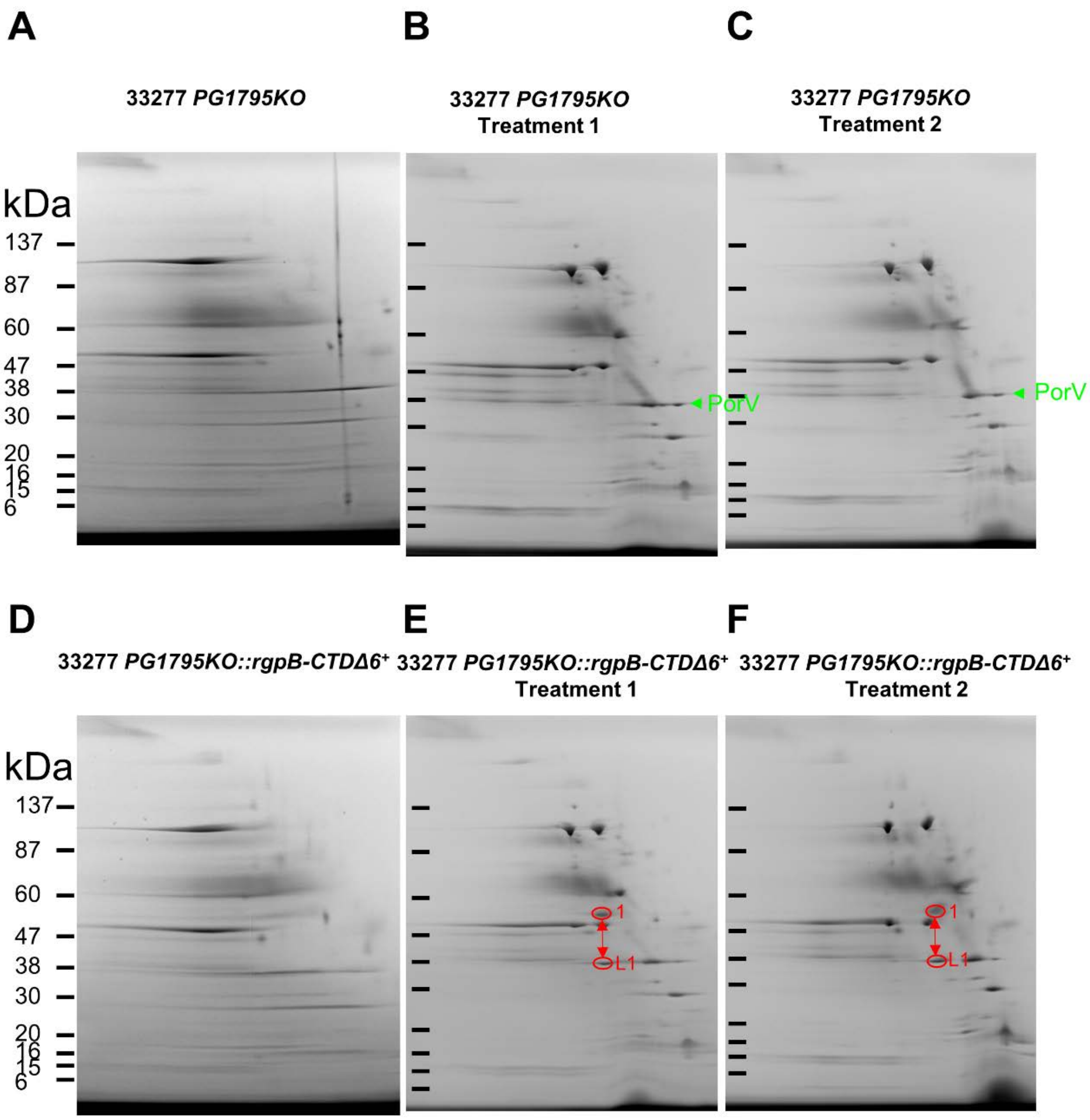

Supplementary Fig. S7. The immature RgpB form IV of the CTD-cleavage mutant RgpB-CTDΔ6 interacts with PorV. Coomassie stained 2D BN-PAGE analyses of OMV lysates from 33277 PG1795KO (**A**, **B**, **C**) and 33277 *PG1795KO::rgpB-CTDΔ6*$^{+}$ (**D**, **E**, **F**) with no pre-treatment of lysate (**A** and **D**) or with lysate pre-treatment 1 (**B** and **E**) or lysate pre-treatment 2 (**C** and **F**). Protein spots indicated in (E) and (F) were analysed by MS (Supplementary Table S5).

References

Glew, M. D., P. D. Veith, D. Chen, C. A. Seers, Y. Y. Chen and E. C. Reynolds (2014). "Blue native-PAGE analysis of membrane protein complexes in *Porphyromonas gingivalis*." Journal of Proteomics **110**: 72-92.

Supplementary Table S1: MS data for Fig. 1 and Supplementary Fig. S1.

Spot #	Data type	Locus tag	Protein name	Mascot Score	Expect Value	Protein Mass (kDa)	# peptide matches
1	MALDI TOF	PG0026	PorU	171	1.80E-14	128.6	26
2	MALDI TOF	PG1604	PorZ	113	1.10E-08	84.1	17
3	MALDI TOF	PG0027	PorV	95	6.90E-07	43.2	7
4	MALDI TOF	PG0602	PorQ	49	0.027	38.2	4
5	MALDI TOF	PG0026	PorU	107	4.50E-08	128.6	26
6	MALDI TOF	PG1604	PorZ	91	1.70E-06	84.1	14
7	MALDI TOF	PG0027	PorV	68	0.00035	43.2	12
8	MALDI TOF	PG0602	PorQ	57	0.0048	38.2	5
9	MALDI TOF	PG0026	PorU	93	1.00E-06	128.6	23
10	MALDI TOF	PG1604	PorZ	56	0.0059	84.1	14
11	MALDI TOF	PG0027	PorV	54	0.0085	43.2	9
12	MALDI TOF	PG0602	PorQ	42	0.14	38.2	3
13	LC-MS/MS	PGN_0509	PorZ	111	0.0000018*	84.1	5
14	LC-MS/MS	PGN_0645	PorQ	84	0.0000016*	38.2	4

*Expect value shown is the expect value of the most confidently identified peptide.

Supplementary Table S2: Quantitation of Coomassie stain intensities of complex components from glycerol gradient fractionated OMV lysates separated by 2D BN-PAGE.

**Strain:**	W50				W50 ABK, A-LPS^{-}		*porU$_{C690A}$*	
**Complex Components:**	PorU	PorV	PorZ	PorQ	PorU	PorV	PorZ	PorQ
**Stoichiometry:**	1.3	3.4	1.0	1.1	1.0	1.5	1.3	1.0
**Calculated Monomer Size (kDa)**	126	40.2	80.9	33.9	126	40.2	80.9	33.9
**Observed Complex Size (kDa):**	440				330		200	

The stoichiometry of the PorU : PorV subcomplex from *P. gingivalis* strain W50 ABK, A-LPS^{-} was calculated from a previously produced 2D BN-PAGE (Glew et al. 2014).

Glew MD, Veith PD, Chen D, Seers CA, Chen YY, Reynolds EC. 2014. Blue native-PAGE analysis of membrane protein complexes in *Porphyromonas gingivalis*. J Proteomics. 110*:*72-92.

Supplementary Table S3: MS data for Fig. 5A.

(A) Summary Data

Fraction #	Spot #	Data type	Locus tag	Protein name	Mascot Score	Expect Value	Protein Mass (kDa)	# Peptide matches
F5	3	MALDI-TOF	PG2102	TapA	48	0.035	61.3	5
F5	4	MALDI-TOF	PG2172	PG2172	53	0.012	26.9	6
F5	L3	MALDI-TOF	PG0027	PorV	50	0.024	43.2	8
F5	L4	MALDI-TOF	PG0027	PorV	82	1.50E-05	43.2	11
F6	1	LC-MS/MS	PG1837	HagA	58	0.0023*	285.0	3
F6	2	LC-MS/MS	PG1844	Kgp	127	6.10E-05*	188.7	6
F6	2	LC-MS/MS	PG2024	RgpA	93	0.00018*	186.8	3
F6	3	MALDI-TOF	PG2102	TapA	64	0.00095	61.3	8
F6	4	MALDI-TOF	PG2172	PG2172	69	0.00031	26.9	5
F6	L1	MALDI-TOF	PG0027	PorV	44	0.1	43.2	5
F6	L2	MALDI-TOF	PG0027	PorV	103	1.10E-07	43.2	11
F6	L3	MALDI-TOF	PG0027	PorV	88	3.30E-06	43.2	11
F6	L4	MALDI-TOF	PG0027	PorV	123	1.10E-09	43.2	15

*Expect value shown is the expect value of the most confidently identified peptide.

(B) Peptides matched to T9SS substrates

	Fraction	Mass (Exp)	Mass (Calc)	Mass error	Peptide sequence[1]
**TapA (spot 3)**	F5	1620.8694	1620.8334	0.0360	IWSVELMNKPGGYK
	F5	2650.2834	2650.2915	-0.0081	LVYKEDFSNVQLYDVDIASNYR
	F5	1848.9006	1848.9345	-0.0340	GLVCWSWVEYLPGKR
	F5	3338.7544	3338.6427	0.1117	LYPNPAQEYAVISLPTAANCKAVVYDMQGR
	F5	2331.1880	2331.1971	-0.0091	VVAEASFSGNEYRLNVQHLAK
	F6	1178.6370	1178.6044	0.0326	DQGATYQKLR
	F6	2650.2865	2650.2915	-0.0050	LVYKEDFSNVQLYDVDIASNYR
	F6	2146.9904	2146.9807	0.0097	EDFSNVQLYDVDIASNYR
	F6	1149.6073	1149.5666	0.0407	NLLFSQDGEK
	F6	1306.7002	1306.6669	0.0333	NANHYLITYAK
	F6	1287.6722	1287.6360	0.0362	WANYDKIHNK
	F6	1427.7078	1427.6681	0.0397	VVAEASFSGNEYR
	F6	1307.6813	1307.6721	0.0091	VVSDTERFVEK

**PG2172 (spot 4)**	F5	3729.7271	3729.7410	-0.0139	FPVSNEGHTGAFTFSCFPESGAPGTELATVNINFK
	F5	1462.7992	1462.7892	0.0099	YKGGGTGLTNIGLGR
	F5	1171.6546	1171.6310	0.0237	GGGTGLTNIGLGR
	F5	1486.8905	1486.8871	0.0034	RLALEVYNLLGVK
	F5	1330.7833	1330.7860	-0.0027	LALEVYNLLGVK
	F5	1878.9889	1878.9840	0.0049	VFTSQLPAGSGSYTLPVR
	F6	1462.7865	1462.7892	-0.0028	YKGGGTGLTNIGLGR
	F6	1171.6321	1171.6310	0.0011	GGGTGLTNIGLGR
	F6	1486.8713	1486.8871	-0.0159	RLALEVYNLLGVK
	F6	1330.7695	1330.7860	-0.0166	LALEVYNLLGVK
	F6	1878.9666	1878.9840	-0.0174	VFTSQLPAGSGSYTLPVR

	**Mass (Exp)**	**Charge**	**Mass (Calc)**	**Mass error**	**Mascot Score**	**Expect Value**	**Peptide sequence[1]**
HagA (spot 1)	1473.8654	2	1473.7715	0.0939	25.2	0.027	IDLSPDNYLVTPK
	1055.7054	2	1055.5975	0.1079	29.8	0.012	TVVTAPEAIR
	2090.1082	3	2090.0433	0.0649	35.7	0.0023	FGLSTEADGAKPQSVWIER
Kgp (spot 2)	2012.1382	3	2011.9639	0.1742	17.6	0.15	SDDPEKVPFVYNAAAYAR
	2069.2882	3	2069.1521	0.1361	48.4	0.0001	IAALTINPVQYDVVANQLK
	1763.8282	3	1763.8115	0.0167	21.7	0.047	DVYTDHGDLYNTPVR
	1621.8054	2	1620.8107	0.9947	38.4	0.0012	QITENGNYDVVITR
	1714.9254	2	1714.889	0.0364	36.2	0.002	IGDGLFVTIEPANDVR
	1371.6654	2	1371.6354	0.0301	51.8	6.10E-05	MWIAGDGGNQPAR
	2090.1382	3	2090.0433	0.0949	46.7	0.00018	FGLSTEADGAKPQSVWIER
	1714.9254	2	1714.889	0.0364	36.2	0.002	IGDGLFVTIEPANDVR
RgpA (spot 2)	1371.6654	2	1371.6354	0.0301	51.8	6.10E-05	MWIAGDGGNQPAR

[1]Peptide sequences in green belong to pro domains, sequences in blue belong to CTD regions

Supplementary Table S4: MS data for Fig. 5B.

(A) Summary Data

Fraction #	Spot #	Data type	Locus tag	Protein name	Mascot Score	Protein Expect	Protein Mass (kDa)	# Peptide matches
F5	2	MALDI-TOF	PGN_1728	Kgp	84	9.10E-06	188.7	22
F5	2	MALDI-TOF	PGN_1970	RgpA	53	0.011	186.8	22
F5	3	MALDI-TOF	PGN_1466	RgpB	73	0.00011	81.3	8
F5	4	LC-MS/MS	PGN_0123	PG2172	67	0.00018*	26.9	2
F5	L2	MALDI-TOF	PGN_0023	PorV	87	4.90E-06	43.2	7
F5	L3	MALDI-TOF	PGN_0023	PorV	109	2.80E-08	43.2	13
F5	L4	MALDI-TOF	PGN_0023	PorV	109	2.80E-08	43.2	12
F6	2	MALDI-TOF	PGN_1728	Kgp	126	5.60E-10	188.7	26
F6	2	MALDI-TOF	PGN_1970	RgpA	34	0.91	186.8	17
F6	3	MALDI-TOF	PGN_1466	RgpB	65	0.00077	81.3	13
F6	4	LC-MS/MS	PGN_0123	PG2172	98	0.0002*	26.9	5
F6	L2	MALDI-TOF	PGN_0023	PorV	145	7.10E-12	43.2	14
F6	L3	MALDI-TOF	PGN_0023	PorV	146	5.60E-12	43.2	16
F6	L4	MALDI-TOF	PGN_0023	PorV	127	4.50E-10	43.2	14

*Expect value shown is the expect value of the most confidently identified peptide.

(B) Peptides matched to T9SS substrates

	Fraction	Mass (Exp)	Mass (Calc)	Mass error	Peptide sequence[1]
**Kgp (Spot 2)**	F5	1825.8495	1825.8629	-0.0134	LDAPTTRTTCTNNSFK
	F5	2585.3645	2585.3126	0.0519	GGTFASVSIPGAFPTGEVGSPEVPAVR
	F5	2106.0909	2106.0269	0.0640	VKSFTEQVYSLNQYGSEK
	F5	2012.0283	2011.9639	0.0644	SDDPEKVPFVYNAAAYAR
	F5	1340.7009	1340.6877	0.0132	VPFVYNAAAYAR

	F5	2055.0718	2055.0129	0.0589	KGFVGQELTQVEMLGTMR
	F5	2119.0926	2119.0182	0.0744	NNIEIEVSFQGADEVATQR
	F5	1800.8787	1800.8246	0.0541	LYDASFSPYFETAYK
	F5	1763.8579	1763.8115	0.0465	DVYTDHGDLYNTPVR
	F5	2695.3073	2695.2152	0.0921	KVTDLYYSAVDGDYFPEMYTFR
	F5	2567.2282	2567.1203	0.1079	VTDLYYSAVDGDYFPEMYTFR
	F5	2429.1911	2429.1705	0.0206	MSASSPEELTNIIDKVLMYEK
	F5	1871.0479	1870.9901	0.0578	RIGDGLFVTIEPANDVR
	F5	1714.9444	1714.8890	0.0554	IGDGLFVTIEPANDVR
	F5	1387.6805	1387.6303	0.0502	MWIAGDGGNQPAR
	F5	1319.6216	1319.6034	0.0183	YDDFTFEAGKK
	F5	833.3970	833.3742	0.0228	YTFTMR
	F5	2552.2598	2552.2046	0.0552	VKYYYAVNDGFPGDHYAVMISK
	F5	2325.1150	2325.0412	0.0738	YYYAVNDGFPGDHYAVMISK
	F5	2210.1689	2210.0492	0.1198	TGTNAGDFTVVFEETPNGINK
	F5	3054.4905	3054.4128	0.0777	IKEGLTETTFEEDGVATGNHEYCVEVK
	F5	1549.7931	1549.8617	-0.0686	TVQLPAGTKYVAFR
**RgpA (Spot 2)**	F5	1549.7931	1549.7810	0.0121	MDNLKFTEVQTPK
	F5	1390.6846	1390.6517	0.0328	GTFAGFEDTYKR
	F5	1331.6418	1331.5751	0.0667	RMFMNYEPGR
	F5	2210.1689	2210.1583	0.0106	EGNDLTYVLLIGDHKDIPAK
	F5	2409.1943	2409.1349	0.0594	SDQVYGQIVGNDHYNEVFIGR
	F5	1389.6957	1389.7326	-0.0369	IIKCYDPGVTPK
	F5	2500.3002	2500.2744	0.0258	DGKPTGTVAIIASTINQSWASPMR
	F5	1848.9950	1848.9444	0.0506	MLDTWTVFGDPSLLVR
	F5	2552.2598	2552.2046	0.0552	VKYYYAVNDGFPGDHYAVMISK
	F5	2325.1150	2325.0412	0.0738	YYYAVNDGFPGDHYAVMISK
	F5	2090.1096	2090.0433	0.0664	FGLSTEADGAKPQSVWIER
	F5	3054.4905	3054.4128	0.0777	IKEGLTETTFEEDGVATGNHEYCVEVK
	F5	1866.9501	1866.9476	0.0025	AQPDGGDVVLKWEAPSAK
	F5	904.4842	904.4614	0.0228	TEGSREVK

	F5	1871.0479	1870.9901	0.0578	RIGDGLFVTIEPANDVR
	F5	1714.9444	1714.8890	0.0554	IGDGLFVTIEPANDVR
	F5	1387.6805	1387.6303	0.0502	MWIAGDGGNQPAR
	F5	1319.6216	1319.6034	0.0183	YDDFTFEAGKK
	F5	833.3970	833.3742	0.0228	YTFTMR
	F5	1309.6961	1309.6878	0.0084	IKEGLTETTYR
	F5	1825.8495	1825.7611	0.0884	DAGMSAQSHEYCVEVK
	F5	2807.4872	2807.4416	0.0457	VCVDYIPDGVADVTAQKPYTLTVVGK
**RgpB (Spot 3)**	F5	1483.7321	1483.6977	0.0344	MDNLQFTGVQTSK
	F5	841.5155	841.4909	0.0246	DVLIAPSK
	F5	2000.0726	2000.0578	0.0148	VAEDIASPVTANAIQQFVK
	F5	1671.8398	1671.8468	-0.0070	EGNDLTYVLLVGDHK
	F5	2409.1683	2409.1349	0.0333	SDQVYGQIVGNDHYNEVFIGR
	F5	819.4225	819.3974	0.0251	NITTEDK
	F5	1035.5010	1035.4695	0.0315	CYDPGVTPK
	F5	1766.7832	1766.7678	0.0154	TFGGVTMNGMFAMVEK
**Kgp (Spot 2)**	F6	1013.5803	1013.5869	-0.0066	IKLDAPTTR
	F6	2585.4204	2585.3126	0.1077	GGTFASVSIPGAFPTGEVGSPEVPAVR
	F6	1290.8534	1290.8024	0.0510	LIAVPVGATPVVR
	F6	2106.1386	2106.0269	0.1117	VKSFTEQVYSLNQYGSEK
	F6	1878.9787	1878.8636	0.1152	SFTEQVYSLNQYGSEK
	F6	2012.0500	2011.9639	0.0861	SDDPEKVPFVYNAAAYAR
	F6	1340.7427	1340.6877	0.0550	VPFVYNAAAYAR
	F6	2055.0878	2055.0129	0.0749	KGFVGQELTQVEMLGTMR
	F6	1927.0044	1926.9180	0.0865	GFVGQELTQVEMLGTMR
	F6	2069.2386	2069.1521	0.0866	IAALTINPVQYDVVANQLK
	F6	2119.1246	2119.0182	0.1064	NNIEIEVSFQGADEVATQR
	F6	1800.9087	1800.8246	0.0840	LYDASFSPYFETAYK
	F6	1763.8961	1763.8115	0.0846	DVYTDHGDLYNTPVR
	F6	2692.4844	2692.3596	0.1248	YNDGLAASAAPVFLALVGDTDVISGEK

	F6	2567.2523	2567.1203	0.1320	VTDLYYSAVDGDYFPEMYTFR
	F6	2429.2415	2429.1705	0.0710	MSASSPEELTNIIDKVLMYEK
	F6	1620.8897	1620.8107	0.0790	QITENGNYDVVITR
	F6	1870.8198	1870.9901	-0.1703	RIGDGLFVTIEPANDVR
	F6	1714.9733	1714.8890	0.0843	IGDGLFVTIEPANDVR
	F6	1387.7037	1387.6303	0.0734	MWIAGDGGNQPAR
	F6	1191.5769	1191.5084	0.0684	YDDFTFEAGK
	F6	1319.6634	1319.6034	0.0600	YDDFTFEAGKK
	F6	2325.1510	2325.0412	0.1098	YYYAVNDGFPGDHYAVMISK
	F6	2210.2087	2210.0492	0.1595	TGTNAGDFTVVFEETPNGINK
	F6	2089.0884	2089.0592	0.0291	FGLSTEANGAKPQSVWIER
	F6	1549.8438	1549.8617	-0.0179	TVQLPAGTKYVAFR
**RgpA (Spot 2)**	F6	1549.8438	1549.7810	0.0628	MDNLKFTEVQTPK
	F6	1838.8536	1838.8720	-0.0184	GMAQVPTYTEGVNLSEK
	F6	1850.0443	1849.9614	0.0829	FFPGEIATLDDPFILR
	F6	1390.7221	1390.6517	0.0704	GTFAGFEDTYKR
	F6	1331.6987	1331.5751	0.1237	RMFMNYEPGR
	F6	1319.6634	1319.6470	0.0165	YTPVEEKQNGR
	F6	2210.2087	2210.1583	0.0504	EGNDLTYVLLIGDHKDIPAK
	F6	2409.2407	2409.1349	0.1057	SDQVYGQIVGNDHYNEVFIGR
	F6	1653.0133	1652.9349	0.0784	ELVLLSVSDAPELLR
	F6	981.5887	981.5106	0.0781	YHFLMKK
	F6	2446.2693	2446.1976	0.0717	DVTVEGSNEFAPVQNLTGSAVGQK
	F6	2325.1510	2325.0412	0.1098	YYYAVNDGFPGDHYAVMISK
	F6	1870.8198	1870.9901	-0.1703	RIGDGLFVTIEPANDVR
	F6	1714.9733	1714.8890	0.0843	IGDGLFVTIEPANDVR
	F6	1387.7037	1387.6303	0.0734	MWIAGDGGNQPAR
	F6	1191.5769	1191.5084	0.0684	YDDFTFEAGK
	F6	1309.7069	1309.6878	0.0191	IKEGLTETTYR
**RgpB (Spot 3)**	F6	1483.7349	1483.6977	0.0372	MDNLQFTGVQTSK

F6	1320.6504	1320.6310	0.0194	YTPVEEKENGR
F6	2000.0979	2000.0578	0.0401	VAEDIASPVTANAIQQFVK
F6	2677.3895	2677.3599	0.0297	VAEDIASPVTANAIQQFVKQEYEK
F6	2349.1894	2349.1488	0.0406	QEYEKEGNDLTYVLLVGDHK
F6	2196.1798	2196.1426	0.0372	EGNDLTYVLLVGDHKDIPAK
F6	3018.5845	3018.5199	0.0646	ITPGIKSDQVYGQIVGNDHYNEVFIGR
F6	2409.2087	2409.1349	0.0738	SDQVYGQIVGNDHYNEVFIGR
F6	1116.5869	1116.5775	0.0094	EDLKTQIDR
F6	2562.3306	2562.3177	0.0129	DVKVEGTSIADVANDKPYTVAVSGK
F6	2220.1608	2220.1274	0.0334	VEGTSIADVANDKPYTVAVSGK
F6	2008.0123	2007.9935	0.0187	TITVESPAAGLTIFDMNGR
F6	1752.8332	1752.8730	-0.0397	NRMVFEAQNGVYAVR

		Mass (Exp)	Charge	Mass (Calc)	Mass error	Mascot Score	Expect Value
**PG2172 (Spot 4)**	F5	1879.0882	3	1878.9840	0.1042	46.6	0.00018
	F5	1273.6281	3	1273.7030	-0.0749	40.4	0.00075
**PG2172 (Spot 4)**	F6	1330.8054	2	1330.7860	0.0194	35.7	0.0049
	F6	1878.9255	2	1878.9840	-0.0585	48.0	0.0002
	F6	1879.9582	3	1878.9840	0.9742	32.1	0.011
	F6	1273.7181	3	1273.7030	0.0151	15.3	0.64
	F6	1273.8381	3	1273.7030	0.1351	38.1	0.0034

[1]Peptide sequences in green belong to pro domains, sequences in blue belong to CTD regions

Supplementary Table S5: MS data for Fig. 7E.

(A) Summary Data

Treatment	Spot #	Data type	Locus tag	Protein name	Mascot Score	Protein Mass (kDa)	# Peptide matches
1	1	LC-MS/MS	rRgpB_CTDΔ6	RgpB cleavage mutant	1712	80.8	40
1	1	LC-MS/MS	PGN_1466	RgpB	1252	81.3	27
1	L1	LC-MS/MS	PGN_0023	PorV	2505	43.2	56
2	1	LC-MS/MS	rRgpB_CTDΔ6	RgpB cleavage mutant	976	80.8	27
2	1	LC-MS/MS	PGN_1466	RgpB	832	81.3	23
2	L1	LC-MS/MS	PGN_0023	PorV	1633	43.2	40

(B) Peptides matched to T9SS substrates

	Mass (Exp)	Charge	Mass (Calc)	Mass error	Mascot Score	Expect Value	Peptide sequence[1]
**rRgpB_CTDΔ6**	798.5041	2	798.5037	0.0004	40.79	8.30E-05	MIVIVPK
**(treatment 1)**	814.4983	2	814.4986	-0.0004	43.93	4.00E-05	MIVIVPK
	814.4986	2	814.4986	0	27.31	0.0019	MIVIVPK
	814.4987	2	814.4986	0.0001	22.24	0.006	MIVIVPK
	814.4988	2	814.4986	0.0002	28.68	0.0014	MIVIVPK
	814.4989	2	814.4986	0.0003	15.01	0.032	MIVIVPK
	1714.7721	3	1714.7726	-0.0006	40.56	8.80E-05	KYEEDIEDFVDWK
	1714.7725	2	1714.7726	-0.0001	68.92	1.30E-07	KYEEDIEDFVDWK
	1714.7726	2	1714.7726	0	80.61	8.70E-09	KYEEDIEDFVDWK
	1714.7743	3	1714.7726	0.0017	19.97	0.01	KYEEDIEDFVDWK
	1714.775	2	1714.7726	0.0024	74.13	3.90E-08	KYEEDIEDFVDWK
	1714.7854	2	1714.7726	0.0128	31.18	0.00076	KYEEDIEDFVDWK
	1586.6788	2	1586.6777	0.0011	76.65	2.20E-08	YEEDIEDFVDWK
	2349.1514	3	2349.1488	0.0025	43	5.00E-05	QEYEKEGNDLTYVLLVGDHK
	2349.1524	2	2349.1488	0.0036	105.85	2.60E-11	QEYEKEGNDLTYVLLVGDHK
	2349.1528	4	2349.1488	0.0039	20.84	0.0082	QEYEKEGNDLTYVLLVGDHK
	1671.8477	2	1671.8468	0.0009	68.99	1.30E-07	EGNDLTYVLLVGDHK
	1671.8482	3	1671.8468	0.0014	27.7	0.0017	EGNDLTYVLLVGDHK

	1671.8488	3	1671.8468	0.002	41.01	7.90E-05	EGNDLTYVLLVGDHK
	1671.8492	3	1671.8468	0.0024	48.76	1.30E-05	EGNDLTYVLLVGDHK
	1671.8495	2	1671.8468	0.0027	56.5	2.20E-06	EGNDLTYVLLVGDHK
	2409.1337	3	2409.1349	-0.0012	40.52	8.90E-05	SDQVYGQIVGNDHYNEVFIGR
	2409.1375	2	2409.1349	0.0026	135.1	3.10E-14	SDQVYGQIVGNDHYNEVFIGR
	2409.1388	3	2409.1349	0.0039	77.83	1.60E-08	SDQVYGQIVGNDHYNEVFIGR
	1116.5776	2	1116.5775	0.0001	27.99	0.0016	EDLKTQIDR
	1116.5777	2	1116.5775	0.0002	33.07	0.00049	EDLKTQIDR
	817.4077	2	817.4082	-0.0005	20.52	0.0089	TIHYER
	1766.7692	2	1766.7678	0.0014	55.3	3.00E-06	TFGGVTMNGMFAMVEK
	1864.9422	3	1864.9393	0.0028	52.91	5.10E-06	MLDTWTVFGDPSLLVR
	1864.9429	2	1864.9393	0.0035	98.88	1.30E-10	MLDTWTVFGDPSLLVR
	2193.119	2	2193.1165	0.0025	130.82	8.30E-14	LNESIADETNLTLTVVGYNK
	2193.1195	3	2193.1165	0.0031	71.75	6.70E-08	LNESIADETNLTLTVVGYNK
	900.5644	2	900.5644	0	24.46	0.0036	VTVIKDVK
	2006.0337	3	2006.032	0.0016	42.46	5.70E-05	DVKVEGTNDKPYTVAVSGK
	1663.8426	2	1663.8417	0.0009	81.79	6.60E-09	VEGTNDKPYTVAVSGK
	1663.8434	3	1663.8417	0.0017	51.82	6.60E-06	VEGTNDKPYTVAVSGK
	2007.9927	2	2007.9935	-0.0008	105.49	2.80E-11	TITVESPAAGLTIFDMNGR
	2007.995	3	2007.9935	0.0015	61.05	7.90E-07	TITVESPAAGLTIFDMNGR
	1498.7259	3	1498.7238	0.002	31.67	0.00068	MVFEAQNGVYAVR
	1498.726	2	1498.7238	0.0021	92.68	5.40E-10	MVFEAQNGVYAVR
**Native RgpB- PGN_1466**	1035.4705	2	1035.4695	0.001	50.96	8.00E-06	CYDPGVTPK
**(Unique peptides)**	1480.6168	2	1480.6174	-0.0005	49.24	1.20E-05	GQDEMNEILCEK
**(treatment 1)**	2220.1309	3	2220.1274	0.0035	16.86	0.021	VEGTSIADVANDKPYTVAVSGK
**rRgpB_CTDΔ6**	864.4231	2	864.4229	0.0002	33.47	0.00045	YTPVEEK
**(treatment 2)**	814.4986	2	814.4986	0	31.21	0.00076	MIVIVPK
	814.4987	2	814.4986	0	31.42	0.00072	MIVIVPK
	814.4987	2	814.4986	0.0001	15.22	0.03	MIVIVPK
	1714.7723	2	1714.7726	-0.0003	16.1	0.025	KYEEDIEDFVDWK
	1714.7767	3	1714.7726	0.004	22.62	0.0055	KYEEDIEDFVDWK
	1586.6791	2	1586.6777	0.0014	83.37	4.60E-09	YEEDIEDFVDWK
	801.4706	2	801.4708	-0.0003	19.64	0.011	GLRTEVK
	2349.1534	3	2349.1488	0.0046	47.91	1.60E-05	QEYEKEGNDLTYVLLVGDHK
	1671.8469	3	1671.8468	0.0001	45.52	2.80E-05	EGNDLTYVLLVGDHK

	1671.8475	2	1671.8468	0.0007	65.5	2.80E-07 EGNDLTYVLLVGDHK
	1671.8479	2	1671.8468	0.0011	61.09	7.80E-07 EGNDLTYVLLVGDHK
	1671.8484	3	1671.8468	0.0016	35.71	0.00027 EGNDLTYVLLVGDHK
	1671.8489	2	1671.8468	0.0021	64.12	3.90E-07 EGNDLTYVLLVGDHK
	1671.849	3	1671.8468	0.0022	37.39	0.00018 EGNDLTYVLLVGDHK
	2409.1375	3	2409.1349	0.0025	83.34	4.60E-09 SDQVYGQIVGNDHYNEVFIGR
	1116.5774	2	1116.5775	-0.0001	24	0.004 EDLKTQIDR
	817.4083	2	817.4082	0.0001	16.89	0.02 TIHYER
	1864.9497	2	1864.9393	0.0104	57.21	1.90E-06 MLDTWTVFGDPSLLVR
	2193.1181	3	2193.1165	0.0016	49.51	1.10E-05 LNESIADETNLTLTVVGYNK
	2193.1188	2	2193.1165	0.0024	150.18	9.60E-16 LNESIADETNLTLTVVGYNK
	900.5643	2	900.5644	-0.0001	50.38	9.20E-06 VTVIKDVK
	1663.842	3	1663.8417	0.0003	53.34	4.60E-06 VEGTNDKPYTVAVSGK
	1663.8428	2	1663.8417	0.0011	76.23	2.40E-08 VEGTNDKPYTVAVSGK
	2007.9959	3	2007.9935	0.0024	35.91	0.00026 TITVESPAAGLTIFDMNGR
	2007.9961	2	2007.9935	0.0026	52.27	5.90E-06 TITVESPAAGLTIFDMNGR
	1498.7249	2	1498.7238	0.0011	54.76	3.30E-06 MVFEAQNGVYAVR
**Native RgpB- PGN_1466 (Unique peptides) (treatment 2)**	808.3752	2	808.3756	-0.0004	20.24	0.0095 DFVDWK
	756.3054	2	756.3112	-0.0058	19.43	0.011 FSCESK
	1035.4704	2	1035.4695	0.0008	44.32	3.70E-05 CYDPGVTPK
	1480.6183	2	1480.6174	0.0009	49.35	1.20E-05 GQDEMNEILCEK

[1]Peptide sequences in blue belong to CTD regions, sequences in red contain the RgpB-CTDΔ6 deletion

Supplementary Table S6: MaxQuant data.

(A) Components of the attachment and translocation complexes - strain W50

Locus Tag	Name	Unique peptides W50_R1	Unique peptides W50_R2	Unique peptides W50_R3	MW [kDa]	Q-value	Score	Sequence coverage W50_R1 [%]	Sequence coverage W50_R2 [%]	Sequence coverage W50_R3 [%]	iBAQ W50_R1	iBAQ W50_R2	iBAQ W50_R3	MS/MS Count W50_R1	MS/MS Count W50_R2	MS/MS Count W50_R3
PG0026	PorU	25	29	28	128.3	0	147.7	31.3	38.7	37.9	4.18E+07	4.55E+07	4.29E+07	32	28	25
PG0027	PorV	21	20	22	43.3	0	323.3	71.6	69.1	81.8	7.28E+08	7.33E+08	7.64E+08	39	34	38
PG0288	PorK	18	19	19	55.9	0	151.2	42.6	49.1	49.1	1.01E+08	9.45E+07	8.86E+07	22	18	20
PG0289	PorL	16	17	17	39.3	0	323.3	61.6	61.6	61.6	3.14E+08	2.94E+08	3.06E+08	23	24	31
PG0290	PorM	17	17	18	56.5	0	201.0	40.9	40.9	44.2	9.84E+07	1.04E+08	1.07E+08	26	24	20
PG0291	PorN	13	13	13	41.7	0	158.6	41.8	39.9	39.9	1.02E+08	9.87E+07	9.49E+07	12	14	13
PG0602	PorQ	6	8	9	38.1	0	74.6	28	46	52.3	1.93E+07	3.03E+07	3.08E+07	5	6	7
PG1604	PorZ	17	20	18	83.7	0	136.3	32.1	37.1	34	4.84E+07	5.32E+07	5.43E+07	18	17	15

(B) T9SS substrates - strain W50

Locus Tag	Name	Unique peptides W50_R1	Unique peptides W50_R2	Unique peptides W50_R3	MW [kDa]	Q-value	Score	Sequence coverage W50_R1 [%]	Sequence coverage W50_R2 [%]	Sequence coverage W50_R3 [%]	iBAQ W50_R1	iBAQ W50_R2	iBAQ W50_R3	MS/MS Count W50_R1	MS/MS Count W50_R2	MS/MS Count W50_R3
PG0182	PG0182	4	5	6	134.7	0	309.1	9.3	11.8	10.5	2.96E+06	2.68E+06	3.04E+06	3	5	4
PG0183	PG0183	9	15	14	240.1	0	43.0	8.2	11.5	9.2	1.98E+06	3.71E+06	2.86E+06	4	12	9
PG0232	CPG70	30	30	30	91.5	0	323.3	43.7	40.4	40.7	3.68E+08	3.49E+08	3.44E+08	44	41	44
PG0350	PG0350	11	12	12	52.7	0	192.7	30	34.1	34.1	1.46E+08	1.05E+08	1.54E+08	14	13	15
PG0411	PG0411	12	12	12	103.6	0	62.2	18.3	18.8	17.8	3.52E+07	3.33E+07	3.68E+07	12	12	14
PG0495	PG0495	9	10	11	53.9	0	39.7	24.3	26.2	29	9.63E+06	9.03E+06	8.92E+06	6	6	6
PG0506	RgpB	26	30	28	81.0	0	323.3	67	82.9	80.3	6.72E+08	6.64E+08	7.18E+08	52	54	56
PG0553	PG0553	37	37	37	102.5	0	323.3	59	59.4	59	2.65E+08	2.35E+08	2.37E+08	56	53	55
PG0611	PG0611	4	4	5	35.7	0	32.4	17.4	17.4	23.1	2.77E+07	3.67E+07	3.59E+07	4	7	5
PG0614	PG0614	3	3	3	36.6	0	12.1	7.7	7.7	10.1	6.77E+06	7.72E+06	5.49E+06	2	2	3
PG0616	HBP35	20	21	21	37.6	0	323.3	70.3	70.6	61.6	8.83E+08	8.67E+08	8.78E+08	40	38	40
PG0626	PG0626	1	2	1	32.5	0	16.7	10.8	16.3	10.8	1.85E+06	1.78E+06	1.85E+06	1	2	1
PG0654	PG0654	11	14	14	44.9	0	153.4	21.5	40.5	40.5	1.76E+08	1.62E+08	1.66E+08	16	18	16
PG1030	PG1030	6	6	6	50.1	0	89.4	20.4	20	19.1	2.20E+07	2.39E+07	2.07E+07	5	6	6
PG1374	PG1374	12	13	13	47.1	0	144.2	35	38.8	38.8	1.04E+08	1.09E+08	1.04E+08	13	15	15
PG1424	PAD	35	37	37	61.7	0	323.3	71.2	73.4	73.4	1.27E+09	1.16E+09	1.35E+09	84	89	93
PG1427	PG1427	11	11	10	93.1	0	133.4	22.4	22.7	20.9	2.35E+07	1.91E+07	2.02E+07	13	10	11
PG1548	PG1548	15	14	14	93.4	0	168.4	27.4	25.6	25.6	5.61E+07	5.80E+07	5.91E+07	24	19	21
PG1795	PG1795	7	7	7	28.9	0	95.6	48.7	48.7	48.7	6.21E+08	1.05E+09	8.88E+08	19	29	19
PG1798	PG1798	11	12	12	45.7	0	206.1	41	41	47.9	5.35E+07	5.13E+07	5.40E+07	13	15	13
PG1837	HagA	15	13	14	233.4	0	155.3	49.1	46.1	46	8.17E+07	8.24E+07	8.08E+07	17	18	21
PG1844	Kgp	37	38	38	187.8	0	323.3	58.1	59.5	59	1.36E+09	1.52E+09	1.60E+09	142	141	131
PG1969	PG1969	4	4	4	33.3	0	25.2	19	19	19	1.81E+07	1.64E+07	1.57E+07	3	6	3
PG2024	RgpA	40	40	40	185.7	0	323.3	57.5	56.6	56	3.88E+09	3.80E+09	4.13E+09	257	273	262
PG2100	PG2100	17	15	14	63.1	0	177.2	41.3	35.7	32.1	6.17E+07	5.30E+07	5.16E+07	25	28	22
PG2102	TapA	29	26	28	61.1	0	323.3	71.1	67	74.4	1.78E+09	1.65E+09	1.72E+09	72	63	69
PG2172	PG2172	5	7	7	26.6	0	113.2	38.3	49.6	56.9	1.44E+08	1.31E+08	1.36E+08	6	9	9
PG2198	PG2198	6	8	8	32.3	0	33.6	36.5	44	45.7	2.85E+07	3.61E+07	3.40E+07	5	9	8
PG2216	PG2216	9	10	10	61.4	0	111.7	28.8	30.2	30.2	7.79E+07	8.50E+07	9.27E+07	11	14	12

C) Components of the attachment and translocation complexes - strain ATCC 33277

Locus Tag	Name	Unique peptides 1	Unique peptides 2	Unique peptides 3	MW [kDa]	Q-value	Score	Sequence coverage 1 [%]	Sequence coverage 2 [%]	Sequence coverage 3 [%]	iBAQ 1	iBAQ 2	iBAQ 3	MS/MS Count 1	MS/MS Count 2	MS/MS Count 3
PGN_0022	PorU	15	17	18	128.3	0	94.7	17.4	20.5	20	1.78E+07	1.71E+07	1.97E+07	15	17	18
PGN_0023	PorV	22	22	22	43.3	0	323.3	81.8	81.8	81.8	5.10E+08	5.04E+08	4.74E+08	39	38	32
PGN_0509	PorQ	15	15	16	82.6	0	144.1	21.6	23.2	25.8	3.43E+07	3.39E+07	3.51E+07	16	16	16
PGN_0645	PorZ	8	7	10	38.1	0	99.0	46	41.9	57.8	1.99E+07	2.11E+07	2.16E+07	4	8	8
PGN_1673	PorN	12	12	12	41.5	0	104.0	30.9	30.9	30.9	8.19E+07	6.59E+07	6.71E+07	16	12	11
PGN_1674	PorM	16	16	15	56.6	0	169.5	40.7	40.7	40.7	7.53E+07	6.87E+07	6.53E+07	20	18	24
PGN_1675	PorL	16	17	16	34.9	0	323.3	68.9	68.9	68.9	2.35E+08	2.37E+08	2.30E+08	29	28	28
PGN_1676	PorK	18	18	19	55.9	0	158.5	48.3	48.3	48.3	6.92E+07	6.42E+07	6.12E+07	22	20	25

D) T9SS substrates - strain ATCC 33277

Locus Tag	Name	Unique peptides 1	Unique peptides 2	Unique peptides 3	MW [kDa]	Q-value	Score	Sequence coverage 1 [%]	Sequence coverage 2 [%]	Sequence coverage 3 [%]	iBAQ 1	iBAQ 2	iBAQ 3	MS/MS Count 1	MS/MS Count 2	MS/MS Count 3
PGN_0123	PGN_0123	10	9	9	26.5	0	197.4	63.8	63.4	48.8	4.62E+08	4.52E+08	4.47E+08	15	17	9
PGN_0152	TapA	12	11	11	61.2	0	119.0	35.7	30	31.1	2.19E+07	1.76E+07	1.91E+07	11	11	14
PGN_0291	PGN_0291	38	35	36	134.5	0	323.3	45.5	43.4	43.5	9.46E+07	8.98E+07	9.16E+07	59	46	45
PGN_0335	CPG70	32	31	31	91.5	0	323.3	41.9	41.9	40.2	3.36E+08	2.84E+08	2.87E+08	43	39	45
PGN_0654	PGN_0654	8	8	8	35.4	0	88.2	45.7	45.7	45.7	1.30E+08	1.07E+08	1.24E+08	11	8	13
PGN_0657	PGN_0657	5	4	5	34.6	0	17.8	16.9	11.4	14.6	1.55E+07	1.51E+07	1.46E+07	4	4	4
PGN_0659	HBP35	20	20	20	37.6	0	323.3	70.1	70.1	70.1	5.97E+08	7.03E+08	7.01E+08	38	40	39
PGN_0693	PGN_0693	12	12	12	44.9	0	125.8	30.8	30.5	30.5	1.64E+08	1.58E+08	1.48E+08	11	16	20
PGN_0795	PGN_0795	16	15	16	78.6	0	161.2	25.9	23.6	28.3	4.34E+07	4.28E+07	4.11E+07	16	17	13
PGN_0852	PGN_0852	9	8	8	47.1	0	96.3	29.2	24.3	24.3	5.44E+07	5.86E+07	6.11E+07	7	9	10
PGN_0898	PAD	33	34	34	61.7	0	323.3	63.5	71.2	67.4	9.05E+08	8.94E+08	8.73E+08	77	71	82
PGN_0900	PGN_0900	14	12	13	93.2	0	183.3	28.4	24.8	28.1	2.45E+07	2.37E+07	2.40E+07	17	16	17
PGN_1115	PGN_1115	2	2	2	41.1	0	8.9	5.4	5.4	5.4	3.86E+06	4.20E+06	3.50E+06	1	1	1
PGN_1321	PGN_1321	7	6	8	50.1	0	115.3	24.7	19.7	28	2.19E+07	2.31E+07	1.94E+07	8	5	8
PGN_1416	PGN_1416	36	36	34	102.7	0	323.3	57	58.1	53.2	1.80E+08	1.65E+08	1.64E+08	49	47	54

PGN_1466	RgpB	20	20	20	80.9	0	221.3	71.9	71.9	71.1	2.31E+08	2.28E+08	2.37E+08	40	30	41
PGN_1476	PGN_1476	13	13	10	53.3	0	76.9	37.1	37.1	28.2	9.48E+06	8.25E+06	8.17E+06	11	10	6
PGN_1556	PGN_1556	8	8	8	103.6	0	66.8	13.4	13.4	13.4	9.11E+06	1.01E+07	8.15E+06	7	10	5
PGN_1728	Kgp	41	44	41	187.3	0	323.3	59.4	60.1	58.3	1.38E+09	1.39E+09	1.35E+09	144	126	141
PGN_1733	HagA	22	22	23	283.2	0	323.3	45.2	46.9	47.3	2.13E+08	1.98E+08	2.08E+08	37	34	39
PGN_1767	PGN_1767	12	12	13	47.7	0	284.4	43	43	48.7	1.01E+08	9.54E+07	1.12E+08	21	22	23
PGN_1970	RgpA	30	30	29	185.3	0	323.3	56.7	56.7	56	3.35E+09	3.49E+09	3.30E+09	238	255	246
PGN_2080	PGN_2080	9	10	8	61.4	0	127.1	28.8	30.2	24.8	5.57E+07	5.48E+07	5.22E+07	12	11	10

Supplementary Table S7: List of *P. gingivalis* and *E. coli* strains, and *E. coli* plasmids used in this study.

***P. gingivalis* strain**	**Relevant genotype**	**Source**
W50 (ATCC 53978)	Wild type	Reference strain, laboratory collection
ATCC 33277	Wild type	Reference strain, laboratory collection
ATCC 33277*	Wild type	Reference strain, Koji Nakayama laboratory
W50 *porU*	*ΔporU::ermF* (PG0026, HMPREF1322_0007) (Em^r)	(Glew et al. 2012)
33277 *porU*	*ΔporU::ermF* (PGN_0022) (Em^r)	(Glew et al. 2012)
33277 $porU_{C590A}$	$\Delta fimA::porU_{C590A}^{+}$ (Em^r, Tc^r)	(Glew et al. 2012)
33277 *porV*	*porV::cepA* (PGN_0023) ($cepA^+$, Ap^r)	(Chen et al. 2011)
33277 *porQ*	*ΔporQ::ermF* (PGN_0645) (Em^r)	(Sato et al. 2010)
W50 *porZ*	*ΔporZ::ermF* (PG1604, HMPREF1322_1435) (Em^r)	This study
33277 *porZ*	*ΔporZ::ermF* (PGN_0509) (Em^r)	This study
33277 *PG1795KO*	*ΔPG1795::ermF* (PGN_1770) (Em^r)	This study
33277 *PG1795KO::rgpB-CTDΔ6*$^+$	$\Delta PG1795::rgpB_{\Delta(S664\text{-}A669)}^{+}$ ($cepA^+$, Ap^r)	This study
***E. coli* strain**	**Relevant genotype**	**Source**
α–select Gold *Escherichia coli*	F^- *deoR endA1 recA1 relA1 gyrA96 hsdR17*(r_k^-, m_k^+) *supE44 thi-1 pho*AΔ(*lacZYA argF*)U169 Φ80*lac*ZΔM15λ^-	Bioline
BL21-CodonPlus (DE3)-RIPL	*E. coli* B F^- *ompT hsdS*(r_B^- m_B^-) dcm^+ (Tc^r) *gal* λ(DE3) *endA* Hte [*argU proL* (Cm^r)] [*argU ileY leuW* ($Strep/Spec^r$)]	Stratagene
***E. coli* plasmids**	**Relevant features**	**Source**
pΔPG1604-ermF	Plasmid for *porZ* gene deletion mutagenesis, derivative of pGEM-Teasy	This study
pPG1795KO-ErmF	Plasmid for *PG1795* gene deletion mutagenesis, derivative of pGEM-Teasy	This study
pGEM-Teasy	Cloning vector, Ap^r	Promega
pAL30	*ermF* gene ligated into pGEM-Teasy	(Dashper et al. 2009)

pUTKm1	R6K-based suicide plasmid containing the *oriT* (mob) from plasmid RP4	(de Lorenzo et al. 1990), Professor Roy M. Robins-Browne laboratory
pGib1	Derivative of pGEM-Teasy vector with polyclonal site deleted except ApaI and SacI with *ermF* gene inserted between these sites, Ap^r	This study
pGib2	Derivative of pGib1 with *oriT* from pUTKm1 replacing portion of f1 *oriT* between SspI2214 to DraIII2604 (pGEM-Teasy numbering), Ap^r	This study
pIN-ErmF	*cepA* gene from 33277 *porV* strain introduced into the middle (and in same orientation) of the *ermF* gene (at SspI site) of pGib2, Ap^r	This study
pIN-ErmF_rgpB	Introduction of the *rgpB* WT gene from W50 (including promoter) between the upstream region of *ermF* (*ermF_U*) and *cepA* gene in opposite orientation, Ap^r	This study
pIN-ErmF_rgpB-CTDΔ6	Deletion of *rgpB* coding region of CTD cleavage site (S_{664}-A_{669}) of pIN-ErmF_rgpB, Ap^r	This study
pET-30 Ek/LIC	Vector for expression of recombinant His-tagged proteins in *E. coli*, Kan^r	Merck
pPG1604N	Derivative of pET-30 Ek/LIC for expression of N-terminal His-tagged PorZ (N-terminal region Q_{24}-D_{400}), Kan^r	This study

References:

Chen YY, Peng B, Yang Q, Glew MD, Veith PD, Cross KJ, Goldie KN, Chen D, O'Brien-Simpson N, Dashper SG, et al. 2011. The outer membrane protein LptO is essential for the O-deacylation of LPS and the co-ordinated secretion and attachment of A-LPS and CTD proteins in *Porphyromonas gingivalis*. Mol Microbiol. 79(5):1380-1401.

Dashper SG, Ang CS, Veith PD, Mitchell HL, Lo AW, Seers CA, Walsh KA, Slakeski N, Chen D, Lissel JP, et al. 2009. Response of *Porphyromonas gingivalis* to heme limitation in continuous culture. J Bacteriol 191(3)*:*1044-1055.

de Lorenzo V, Herrero M, Jakubzik U, Timmis KN. 1990. Mini-Tn5 transposon derivatives for insertion mutagenesis, promoter probing, and chromosomal insertion of cloned DNA in gram-negative eubacteria. J Bacteriol 172(11)*:*6568-6572.

Glew MD, Veith PD, Peng B, Chen YY, Gorasia DG, Yang Q, Slakeski N, Chen D, Moore C, Crawford S, et al. 2012. PG0026 Is the C-terminal signal peptidase of a novel secretion system of *Porphyromonas gingivalis*. J Biol Chem. 287(29)*:*24605-24617.

Sato K, Naito M, Yukitake H, Hirakawa H, Shoji M, McBride MJ, Rhodes RG, Nakayama K. 2010. A protein secretion system linked to *Bacteroidete* gliding motility and pathogenesis. Proc Natl Acad Sci U S A. 107(1)*:*276-281.

Supplementary Table S8: Primers used in this study.

Name	Sequence
1604U-F-SphI	acgcgcatgcGTCTGCTTATTGTTCACCTCTTTTT
1604U-R-BamHI	acgcggatccAATAAGCCCTGTTGCCTACGATCAA
1604D-F-PstI	acgcctgcagTCTGGATGGGATCGGATATAGGCAT
1604D-R-SacI	acgcgagctcTCCAGTTCGGAAGCTGATCCACTCC
pGEM-Teasy_gF1	gcgatggaAGAGCTCCCAACGCGTTG
pGEM-Teasy_gR1	gctatcggGGGCCCAATTCGCCCTATA
ermF_gF1	ttgggcccCCGATAGCTTCCGCTATTG
ermF_gR1	ggagctctTCCATCGCCAATTTGCCAG
pGEM-T_ermF_gF1	gagagacCACTACGTGAACCATCAC
pGEM-T_ermF_gR1	gaagggcgatatcGAAAAAGGAAGAGTATGAGTATTC
mob_EcoRV_gF1	ctttttcgatatcGCCCTTCCTGGTTGGCTT
mob_DraIII_gR1	ggttcacgtagtgGTCTCTCGCCTGTCCCCT
pGib2_gF1	tgacttgagAATATTCCTTATGGCATTACTTCC
pGib2_gR1	ttggtacccccgggatccTGACACCACTTTGAAAGGaa
cepA_gF1	ggatcccgggggtaccAAAAGAGTTAAGGAAAGTGAAGC
cepA_SspI_gR1	aggaatattCTCAAGTCACCGATAGTGATAG
pGib2v_F1	CCAACGCGTTGGATGCATAGCTTGA
pGib2v_R1	AATTCGCCCTATAGTGAGTCGTATTACA
1795U_F1	gactcactatagggcgaattAGAATCAGTGGGGAGTTCTTTTTCG
1795U_R1	gcggaagctatcgggggcccTCCTCACGAGGTTGCTGTACTG
ermF_F2	GGGCCCCCGATAGCTTCCGC
ermF_R2	GAGCTCTTCCATCGCCAATTTGC
1795D_F1	aattggcgatggaagagctcAATGGTCGTCAGGTATCTCTGTTC
1795D_R1	ctatgcatccaacgcgttggCATCAATGCCCCTATACAAGGC
pGib4v_F1	ggtaccAAAAGAGTTAAGGAAAGTGA
pGib4v_R1	cccgggatccTGACACCACTT
rgpB_F1	taactcttttggtaccCGGCTTCCGTAAAGTCAAAAACATAC
rgpB_R1	gtgtcaggatcccgggGTCTCTTGGCGTAGTGCCAATAATTC
pGib4v_F2	TCACAATTCCACACAACATACGAG
pGib4v_R2	CTCGTATGTTGTGTGGAATTGTGA
rgpB_clDel2_F1	TTATCATTTGTACCTTCCACTTTCAC
rgpB_clDel2_R1	AAGGTACAAATGATAAGCCTTATACTG
1604-N-tagF	GACGACGACAAGATGCAAGCTCAAGAGGAA
1604-N-tagR	GAGGAGAAGCCCGGTTAGTCTCCGTTGGAA

For oligonucleotide sequences, lowercase letters represent Gibson design overlaps, underlined lowercase letters represent non-homologous sequence for introduction of restriction sites and sequence highlighted in yellow represents overlap with pET-30 Ek/LIC vector for ligation-independent cloning.

Supplementary materials and methods

Construction of *P. gingivalis* mutants

All strains and plasmids are listed in Supplementary Fig. S7. *P. gingivalis* mutants were constructed by double crossover recombination using assembled suicide plasmid vectors (described below) that were linearized by restriction enzyme digestion and introduced into *P. gingivalis* strains W50 or ATCC 33277 by electroporation as previously described (Chen et al. 2011). The *porZ* (*PG1604*) delection mutants in W50 and ATCC 33277 were constructed using plasmid pΔPG1604-ermF, and the *PG1795* deletion mutant (33277 *PG1795KO)* was constructed using plasmid pPG1795KO-ErmF and were selected on TSBHI blood agar containing 10 µg/ml erythromycin. The mutant expressing recombinant (33277 *PG1795KO::RgpB-CTDΔ6*$^{+}$, with a 6 amino acid deletion at the CTD cleavage site between S664-A669) was obtained by insertion into the *ermF* cassette of the *PG1796KO* mutant using plasmid pIN-ErmF_rgpB-CTDΔ6 and selection on TSBHI blood agar with 6 µg/ml ampicillin and methicillin.

Construction of suicide plasmid for *porZ* (*PG1604*) deletion mutants

Deletion mutants in *PG1604* (*porZ*) were created by double cross-over recombination using the suicide vector pΔPG1604-ermF that contains upstream and downstream regions of the *PG1604* gene directionally cloned adjacent to an erythromycin resistance cassette (*ermF*). A 385-bp upstream fragment of *PG1604* (1604U) and a 385-bp downstream internal fragment of *PG1604* (1604D) were amplified by PCR using the primer pairs [1604U-F-SphI and 1604U-R-BamHI] and [1604D-F-PstI and 1604D-R-SacI], respectively. The PCR fragment 1604U was then digested with *Bam*HI and *Sph*I and ligated into *Bam*HI and *Sph*I cleaved pAL30 (Dashper et al. 2009), creating plasmid p1604U-ermF. The PCR fragment 1604D was

digested with *Pst*I and *Sac*I and ligated into *Pst*I and *Sac*I cleaved p1604U-ermF, creating pΔPG1604-ermF. Plasmid pΔPG1604-ermF was linearized with *Sca*I and electroporated into *P. gingivalis*.

Construction of suicide plasmids for creating *P. gingivalis* 33277 *PG1795KO* and 33277 *PG1795::rgpB-CTDΔ6*$^{+}$ mutants

Suicide plasmid constructs were assembled from PCR fragments amplified using Q5 Hot Start High Fidelity DNA polymerase (New England Biolabs), purified with AxyPrep PCR Clean-up Kit (Axygen Biosciences), digested with *Dpn*I, if necessary, and then mixed and assembled using Gibson Assembly Master Mix according to the manufacturer's instructions (New England Biolabs). Resulting plasmid clones were screened by restriction enzyme mapping and DNA sequencing.

The pPG1795KO-ErmF suicide plasmid for creating the *PG1795* deletion mutant (*PG1795KO*) was constructed in one step from four PCR fragments: PCR1 was produced from primer pair pGib2v_F1 and pGib2v_R1 plasmid pGib2 as template, PCR2 (PG1795 upstream region) was produced from primer pair 1795U_F1 and 1795U_R1 and using genomic DNA from W50 as template, PCR 3 (ermF cassette) was produced from primers ermF_gF1 and ermF_gR1 using plasmid pAL30 as template, PCR 4 (PG1795 downstream region) was produced from primers 1795D_F1 and 1795D_R1 using genomic DNA from W50 as template.

The pIN-ErmF_rgpB-CTDΔ6 suicide plasmid for creating the mutant expressing recombinant rRgpB-CTDΔ6 in the *PG1795KO* mutant was constructed in five steps. Step 1. Plasmid pGib1 was assembled from two PCR fragments: PCR1 was produced from primer pair

pGEM-Teasy_gF1 and pGEM-Teasy_gR1 using plasmid pGEM-Teasy as template, PCR2 (ermF cassette) was produced from primers ermF_gF1 and ermF_gR1 using plasmid pAL30 as template. Step 2. Plasmid pGib2 was assembled from two PCR fragments: PCR1 was produced from primer pair pGEM-T_ermF_gF1 and pGEM-T_ermF_gR1 using plasmid pGib1 as template, PCR2 was produced from primers mob_EcoRV_gF1 and mob_DraIII_gR1 using plasmid pUTKm1 (de Lorenzo et al. 1990) (GenBank accession number AF102233.1, a kind gift from Professor Roy M. Robins-Browne laboratory, The University of Melbourne). Step 3. Plasmid pIN-ErmF was assembled from two PCR fragments: PCR1 was produced from primer pair pGib2_gF1 and pGib2_gR1 using plasmid pGib2 as template, and PCR2 (cepA ampicillin cassette) was produced from primers cepA_gF1 and cepA_SspI_gR1 using genomic DNA from the 33277 *lptO* mutant as template. Step 4. Plasmid pIN-ErmF_rgpB was assembled from two PCR fragments: PCR1 was produced from primer pair pGib4v_F1 and pGib4v_R1 using pIN-ErmF as template, and PCR2 (*rgpB* gene) was produced from primers rgpB_F1 and rgpB_R1 using genomic DNA from W50 as template. Step 5. Plasmid pIN-ErmF_rgpB-CTDΔ6 was assembled from two PCR fragments: PCR1 was produced from primer pair pGib4v_F2 and rgpB_clDel2_R1 using plasmid pIN-ErmF_rgpB as template, and PCR2 was produced from primers pGib4v_R2 and rgpB_clDel2_F1 also using plasmid pIN-ErmF_rgpB as template.

Construction of PG1604N expression vector

The DNA fragment corresponding to amino acid residues Q_{24}-D_{400} of the *porZ* coding sequence (377 amino acids of N-terminal half of mature protein) was amplified by PCR from *P. gingivalis* W50 genomic DNA using oligonucleotides 1604-N-tagF and 1604-N-tagR (Supplementary Table S8). The amplicon was then cloned into expression vector pET-30

Ek/LIC (Merck) using ligation-independent cloning according to the manufacturer's instructions producing expression vector pPG1604N.

Gingipain activity assays

Arg- and Lys-specific proteolytic activities were determined using synthetic chromogenic substrates N_{α}-benzoyl-L-arginine 4-nitroanilide hydrochloride and N-(p-tosyl)-Gly-Pro-Lys 4-nitroanilide acetate salt (Sigma Aldrich), respectively. Briefly, *P. gingivalis* cultures grown to late log phase were pelleted at 8000g for 10 min. The cell pellet was resuspended in 50 mM Tris-HCl, pH 7.4, 5 mM $CaCl_2$ and 150 mM NaCl (TC150). Using an $OD650_{nm}$ of 0.8 of culture to be equivalent to 2.5 x 10^9 cells per ml, 5.6 x 10^6 cells per 140 µl in TC150 buffer were added per well and then 20 µl of TC150 and 20 µl of 200 mM cysteine, pH 8.0, were added. After the mixture was incubated at 37°C for 10 min, substrate was added to make a final concentration of 1 mM in a final reaction volume of 200 µl per well. The rate of cleavage of the substrate was measured by absorbance at 405 nm at 10 s intervals for a total time of 60 min at 37°C using a PerkinElmer 1420 Multilabel Counter VICTOR3™. The rate of change of absorbance per second was calculated from the gradient of the linear section of the curve obtained by plotting absorbance vs time. Relative rates were compared to WT in triplicate.

References

Chen YY, Peng B, Yang Q, Glew MD, Veith PD, Cross KJ, Goldie KN, Chen D, O'Brien-Simpson N, Dashper SG, et al. 2011. The outer membrane protein LptO is essential for the O-deacylation of LPS and the co-ordinated secretion and attachment of A-LPS and CTD proteins in *Porphyromonas gingivalis*. Mol Microbiol. 79(5)*:*1380-1401.

Dashper SG, Ang CS, Veith PD, Mitchell HL, Lo AW, Seers CA, Walsh KA, Slakeski N, Chen D, Lissel JP, et al. 2009. Response of *Porphyromonas gingivalis* to heme limitation in continuous culture. J Bacteriol 191(3)*:*1044-1055.

de Lorenzo V, Herrero M, Jakubzik U, Timmis KN. 1990. Mini-Tn5 transposon derivatives for insertion mutagenesis, promoter probing, and chromosomal insertion of cloned DNA in gram-negative eubacteria. J Bacteriol 172(11)*:*6568-6572.
